# Robust Inference of Cell-to-Cell Expression Variations from Single- and K-Cell Profiling

**DOI:** 10.1371/journal.pcbi.1005016

**Published:** 2016-07-20

**Authors:** Manikandan Narayanan, Andrew J. Martins, John S. Tsang

**Affiliations:** Systems Genomics and Bioinformatics Unit, Laboratory of Systems Biology, National Institute of Allergy and Infectious Diseases, National Institutes of Health, Bethesda, Maryland, United States of America; Fred Hutchinson Cancer Research Center, UNITED STATES

## Abstract

Quantifying heterogeneity in gene expression among single cells can reveal information inaccessible to cell-population averaged measurements. However, the expression level of many genes in single cells fall below the detection limit of even the most sensitive technologies currently available. One proposed approach to overcome this challenge is to measure random pools of k cells (e.g., 10) to increase sensitivity, followed by computational “deconvolution” of cellular heterogeneity parameters (CHPs), such as the biological variance of single-cell expression levels. Existing approaches infer CHPs using either single-cell or k-cell data alone, and typically within a single population of cells. However, integrating both single- and k-cell data may reap additional benefits, and quantifying differences in CHPs across cell populations or conditions could reveal novel biological information. Here we present a Bayesian approach that can utilize single-cell, k-cell, or both simultaneously to infer CHPs within a single condition or their differences across two conditions. Using simulated as well as experimentally generated single- and k-cell data, we found situations where each data type would offer advantages, but using both together can improve precision and better reconcile CHP information contained in single- and k-cell data. We illustrate the utility of our approach by applying it to jointly generated single- and k-cell data to reveal CHP differences in several key inflammatory genes between resting and inflammatory cytokine-activated human macrophages, delineating differences in the distribution of ‘ON’ versus ‘OFF’ cells and in continuous variation of expression level among cells. Our approach thus offers a practical and robust framework to assess and compare cellular heterogeneity within and across biological conditions using modern multiplexed technologies.

## Author Summary

Different cells can make different amounts of biomolecules such as RNA transcripts of genes. New technologies are emerging to measure the transcript level of many genes in single cells. However, accurate quantification of the biological variation from cell to cell can be challenging due to the low transcript level of many genes and the presence of substantial measurement noise. Here we present a flexible, novel computational approach to quantify biological cell-to-cell variation that can use different types of data, namely measurements directly obtained from single cells, and/or those from random pools of k-cells (e.g., k = 10). Assessment of these different inputs using simulated and real data revealed that each data type can offer advantages under different scenarios, but combining both single- and k-cell measurements tend to offer the best of both. Application of our approach to single- and k-cell data obtained from resting and inflammatory macrophages, an important type of immune cells implicated in diverse diseases, revealed interesting changes in cell-to-cell variation in transcript levels upon inflammatory stimulation, thus suggesting that inflammation can shape not only the average expression level of a gene but also the gene’s degree of expression variation among single cells.

## Introduction

Transcriptomic profiling is widely used in biomedical research, but until recently it often relies on measuring mRNAs pooled from thousands to millions of cells, thus obscuring the well-appreciated biological variation that exists among individual cells of the profiled population. Quantifying variation in gene expression across single cells could help address fundamental biological questions and empower new applications previously not possible using cell-population based measurements. Such new applications include *de novo* assessment of tissue composition without *a priori* knowledge on cell-type defining markers [1,2] and inferring biologically relevant changes in cell-to-cell variations. Despite rapid technological advances, accurate measurement of single-cell expression is a major challenge, particularly because many mRNAs are expressed at levels close to or below the detection limit of current profiling technologies [3,4]. For example, the estimated rate of capturing individual mRNA molecules ranges from ~10% to ~20% using state-of-the-art single-cell RNA-Seq protocols [4,5]. Indeed, typical single-cell gene-expression data obtained by quantitative PCR (qPCR) or RNA-Seq contain a substantial number of zero or non-detected measurements (“non-detects”), which cannot be entirely attributable to cells expressing zero transcripts. For example, some non-detects may arise from technical factors such as measurement noise, and missed capture or amplification of mRNA transcripts at or near the detection limit, as revealed by recent studies using measurements of spike-in standards and statistical inference methods [6–12].

An alternative approach to direct single-cell profiling, called “stochastic profiling” [13], has been proposed to mitigate detection issues: measure the expression of random pools of a small number of cells (k) (e.g., k = 10), followed by computationally deconvolving these pooled-cell measurements to infer the underlying cell-to-cell variation parameters. This approach offers more robust detection due to the increased amount of input mRNA and has been used to, for example, assess whether expression distributions across cells are bimodal [13–15]. Each approach can offer advantages, e.g., single-cell for its direct interpretability and k-cell for improved sensitivity and therefore better quantitative estimates of certain cell-to-cell variation parameters. In principle they can also be complementary, and when both data types are obtained from a cell population, utilizing them together could potentially provide richer information for assessing cellular heterogeneity than using either one alone; however, in practice, no approach has been developed to take advantage of both data types simultaneously.

To utilize both data types jointly and also allow the flexibility of using either one alone, here we present a Bayesian approach (called **QVARKS**) that **q**uantifies the degree and the statistical uncertainty of expression **var**iation across cells by using **k-** and/or **s**ingle-cell data, after accounting for technical detection limits. A key contribution of our approach includes a newly developed statistical model and associated Bayesian inference and model assessment procedures that can handle single-cell, k-cell, or both data types jointly to infer cellular heterogeneity parameters (CHPs), including the fraction of cells in the population expressing the gene (“ON” cells) or variation in expression level among “ON” cells. Both types of cellular heterogeneity can reflect meaningful biology, for example, the former, or “discrete” heterogeneity, may capture the frequency of functionally distinct cell subsets as classically defined by marker gene expression, while the latter, or “continuous” heterogeneity may capture the spread of the expression phenotype that could ultimately influence the overall population-level response to a perturbation [16].

Another feature of QVARKS is that it can model data jointly from two distinct cell populations to quantitatively assess differences in CHPs (or “differential heterogeneity”, DH) between the two conditions. While assessing differences in mean expression between two conditions is widely applied, the biology of differences in cell-to-cell expression variations (CEV) has been underexplored. Given that CEV can play functional roles and can be under genetic regulation [17], QVARKS can be used to help reveal gene expression heterogeneity among cell populations, such as those exposed to different environments or from distinct developmental lineages. QVARKS thus complements existing single-cell data analysis approaches that either focus on identifying differential expressed (DE) genes [7,11,18], or aim to find genes with high overall variability but do not deconvolve the overall variability into discrete vs. continuous components [6,12,10,19].

We systematically assessed the performance of QVARKS using both simulations and joint single- and k-cell data obtained from two biological conditions. We took advantage of QVARKS’ flexibility to handle different input data types to study the relative performance of using single-cell, k-cell or both data types to infer CHPs in a single condition or compare them between two conditions. We found scenarios where different input data types (single-cell, k-cell, or both) offer advantages. However, integrating both single- and k-cell data often offers the advantages of both. We also evaluated whether single-cell data would lead to inferred parameters consistent with k-cell data and vice versa, and found many situations where single- or k-cell analysis on its own led to significantly different results. Thus, this argues for proper integration of the two data types for robust parameter estimation and cross-checking them for consistencies when possible.

We illustrate the practical biological utility of our approach by applying it to compare CEV in resting versus inflammatory-activated human macrophages, an important immune cell type known to function in diverse tissues and biological processes, including chronic inflammation associated with numerous common human diseases and aging [20]. QVARKS revealed significant differences in the CEV of key genes (e.g., *RELA*, a component of NFκB) upon inflammatory activation, potentially reflecting condition-dependent regulation of cellular heterogeneity. QVARKS is provided as an R package with detailed documentation (see Data Availability for download URL), and thus offers a practical, robust approach to quantitatively assess and compare CEV within and among biological states or conditions.

## Results

### QVARKS method overview: Inferring cell-to-cell expression heterogeneity via integrative modeling of single- and/or k-cell measurements

**Fig 1:**
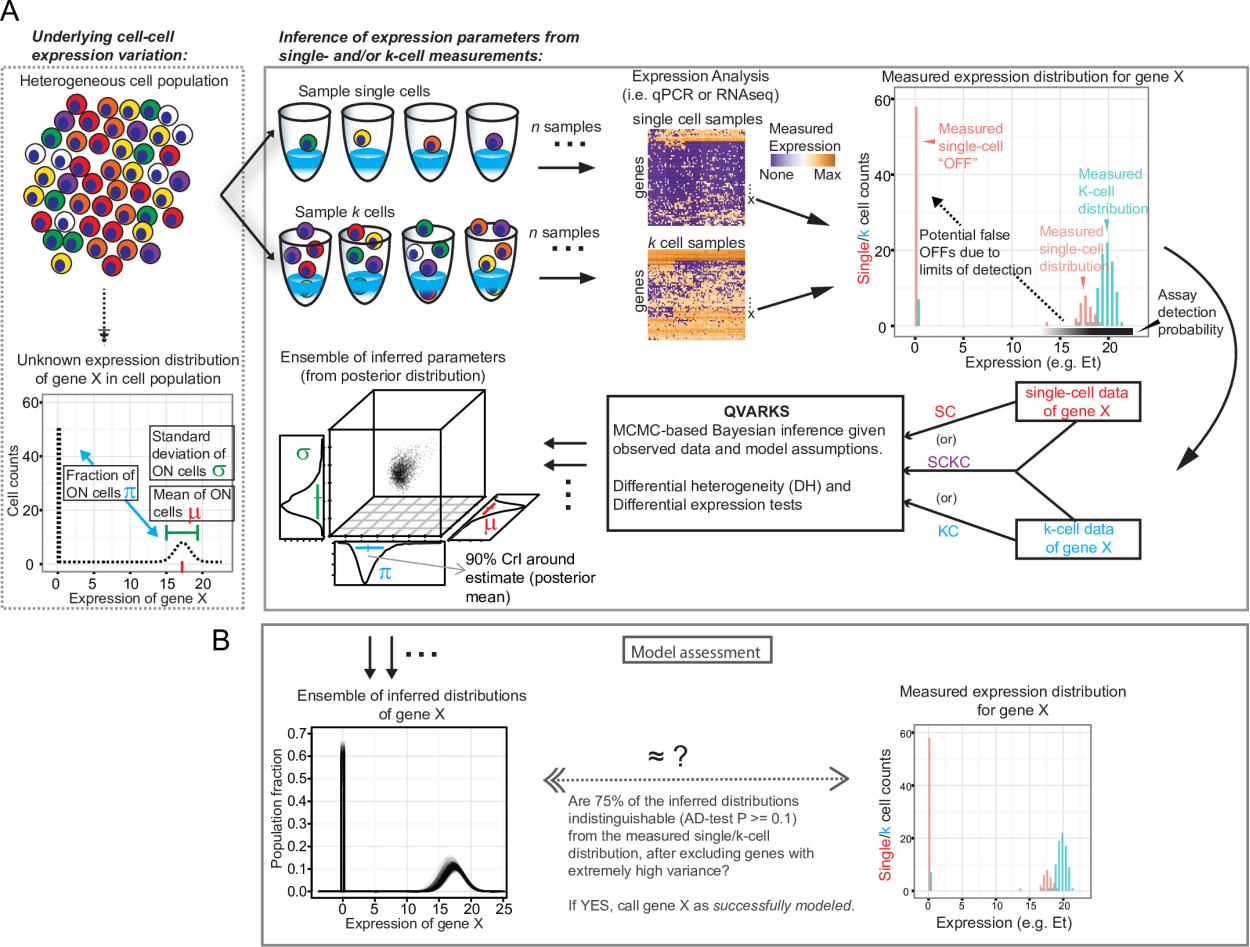
The QVARKS methodology and its three input data modes (SC, KC and SCKC methods). **(A)** Schematic of our experimental and computational strategy (right box) for quantifying cell-to-cell variation in gene expression (left box) by integrating k- and/or single-cell expression profiles is shown (see text for additional details). Our approach can utilize single-cell data alone, or k-cell data alone, or both together as input to infer cellular heterogeneity parameters (CHPs). MCMC stands for Markov Chain Monte Carlo, and our gene expression data is measured in Et = 40-Ct units for qPCR (both in this figure and rest of the paper; please note that Et approximates *log*_2_(transcript counts) as explained in Methods). **(B)** Schematic of our AD-test based model assessment criteria (see text and Methods for details).

We focus on assessing two aspects of CEV for a given gene. First, “discrete” heterogeneity, arises due to the presence of cells with zero (OFF cells) vs. non-zero expression (ON cells) of the gene. Biologically, this type of CEV can originate from differences in the transcriptional status or activity at the gene locus among single cells, e.g., some cells have actively transcribing promoters while others have inactive promoters. Second, “continuous” patterns of CEV among ON cells reflecting, for example, that some cells have higher levels of upstream transcriptional regulators than other cells; inherent stochasticity in biochemical reactions, such as transcript and protein production, can also contribute substantially to continuous variability among single cells [17]. QVARKS mathematically models these two CEV patterns for each gene and the Bayesian procedure described below infers the value of these parameters using single-cell (SC) data alone, k-cell (KC) data alone, or both (SCKC). The overall framework is depicted in Fig 1. The output of QVARKS includes an estimate of the CEV parameters (or their differences between conditions when run in two-condition mode) and the statistical uncertainty around each of the parameters (all computed from the posterior distribution inferred by the Bayesian procedure; see description below and in Methods).

#### Model parameters and assumptions

For each gene we model the distribution of expression level in single cells with two modes: a fraction *π* of cells have at least one transcript and hence are “ON”; the rest of the cells have zero transcripts of this gene and hence are “OFF”. Within ON cells, the level of expression (after logarithmic transformation) is normally distributed with mean *μ* and variance *σ*^2^ (i.e., log-normally distributed) (Fig 1A). To model imperfect transcript detection, such as when the input mRNA amount falls below the detection limit of the assay, we explicitly model the probability of detection (ranging from 0 to 100%) with a mathematical function (logistic function) that assumes higher values as the number of input transcripts increase (see Fig 1A and Methods). To model two distinct cell populations or conditions, each population has its own set of CHPs (*π*, *μ* and *σ*) and we assume the same transcript detection assay is used to profile both conditions. Observed k-cell expression is modeled as a random sampling of k single cells followed by noisy detection as described above (see also Methods). Our choice of statistical distributions used to model single cells is motivated and supported by earlier single-cell modeling studies suggesting log-normal distributions of transcript counts [7,21] and logistic detection behavior of qPCR or RNA-Seq assays [8,11,22], as well as our own data (see bulk DE and model assessments below). However, our overall approach is general and can be extended to alternative choices of single- and k-cell distributions, such as the negative binomial [11,12] and other models of detection limits.

#### Bayesian inference of model parameters

A Bayesian inference procedure (based on a random walk Metropolis Markov Chain Monte Carlo (MCMC) approach) was developed to infer model parameters from SC, KC, or SCKC input modes (Fig 1A). A Bayesian approach was taken to allow the inference of posterior distributions that naturally capture the statistical uncertainty of inferred parameters, so that CHPs within a single cell population or their differences among cell populations can be robustly assessed. For ease of presentation, we summarize each parameter’s posterior distribution by its mean along with a 90% credible interval (CrI) that quantifies the uncertainty or spread around the mean (see Fig 1A and Methods). To assess whether our inferred models capture the data well and thereby also check if the data satisfies our model assumptions and parameterizations, including statistical distribution choices, we verified the agreement between the distribution of observed, real data and of simulated data generated from inferred models, each of which was specified by parameters independently drawn from the posterior distribution [23] (Fig 1B). The specific criterion we used required more than 75% of the models drawn to be capable of generating data samples statistically indistinguishable from the observed single- and/or k-cell data used to infer the parameters (i.e., AD-test P ≥ 0.1 as shown in Fig 1B and Methods), but other model assessment approaches, including a single AD-test approach and posterior predictive checks [23,24] are supported by QVARKS as options (see Discussion). We hereafter refer to inferred models passing model assessment criteria in *all* studied conditions as “successfully modeled” genes, and those passing in a given condition as successfully modeled “gene-condition models” or “gene-condition combinations” (GCCs).

Thus, QVARKS provides a unique, flexible approach to handle three input data modes from the ground up (SC, KC and SCKC), as well as a novel comparative framework to assess changes in heterogeneity parameters, such as *π* and *σ*, between two conditions.

In addition to its ability to integrate single- and k-cell data, QVARKS was designed to provide functionalities that are distinct from and thus complementary to existing single-cell data analysis methods. Approaches such as MAST and SCDE aim to model single cell data to identify differentially expressed (DE) genes among conditions [7,11,18]. The inferred CHPs from QVARKS can also be used to infer DE genes (see below), but the primary goal of QVARKS is to assess CHPs within a single condition or between two conditions. QVARKS also deviates from this class of methods by explicitly modeling: 1) detection drop-offs for each gene to deconvolve the observed fraction of OFF cells (no transcript detected) into those that are truly OFF (transcriptionally silent) vs. technically induced drop-offs, and 2) variation among ON cells to assess continuous patterns of CEV. This type of decomposition is also not yet the focus of other methods such as BASICS that do focus on quantitatively assessing cell-to-cell variability [6,12,10,19]. BASICS, for example, identifies genes with high overall variability unlikely explainable by those originating from technical sources by using a large number of spike-in controls that span the dynamic range of expression to disentangle biological CEV from technical noise. Since QVARKS does not rely on spike-ins, it is applicable to platforms that can only profile a smaller number of genes (e.g., microfluidic based qPCR or targeted RNA-seq).

### Using simulation to compare the performance of SC, KC and SCKC input data modes

We first sought to assess the relative performance of the three input data modes of QVARKS (SC, KC and SCKC) in inferring CHPs (*π*, *μ* and *σ*) across a range of scenarios involving either a single condition/cell population, or two conditions/cell populations aimed at comparing the inferred CHPs between the two conditions. Here, the unique capability of QVARKS for handling all three input data types served as a common inference procedure to help evaluate their relative performance.

**Fig 2:**
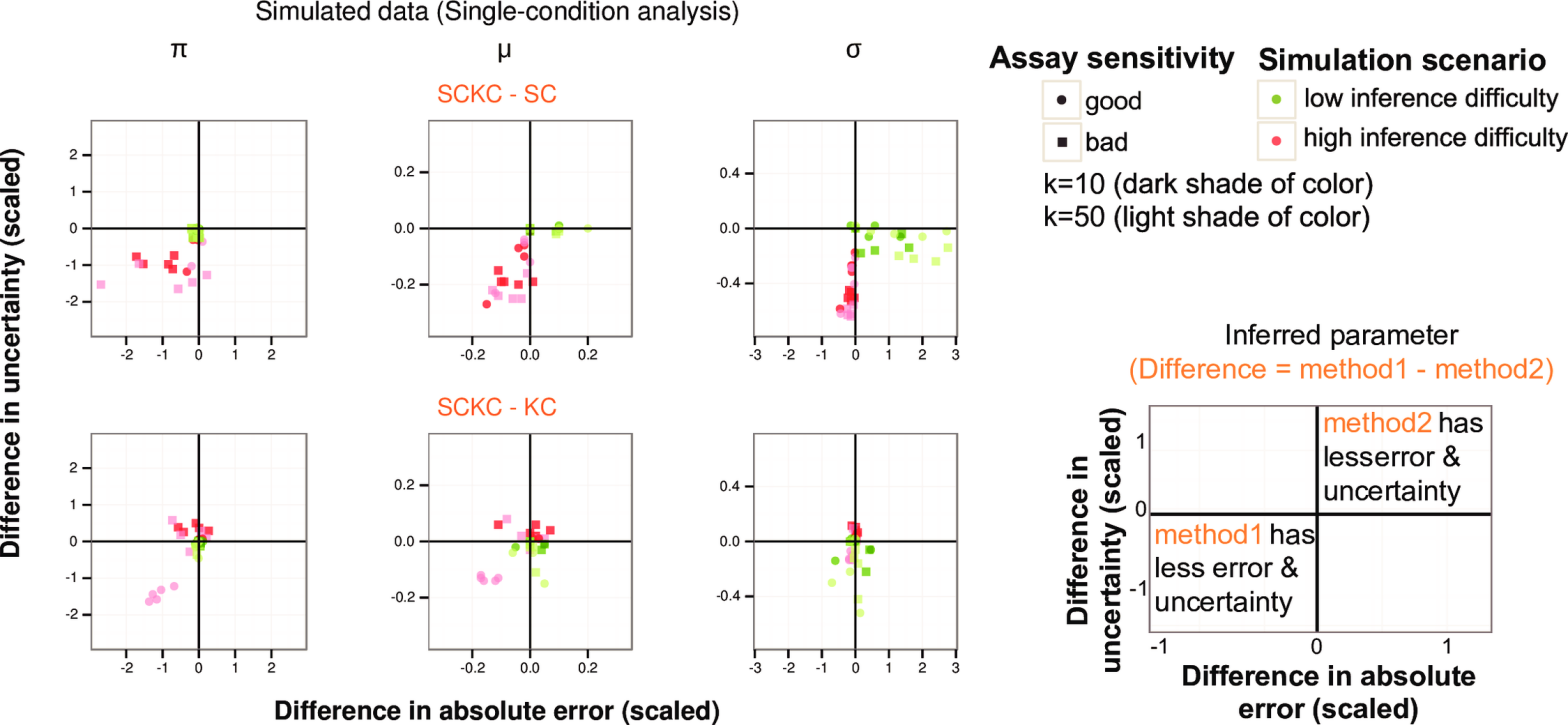
Comparison of SC, KC and SCKC methods using single-condition simulations. Simulation results for single-condition parameter inferences, where each dot corresponds to an inferred parameter for a gene simulated under one condition according to the simulation scenario denoted by the legend. Diverse scenarios of varying levels of inference difficulty, measurement noise and assay sensitivity/detection (see Methods and S1 Fig) were used to generate simulated data. Parameter inferences (posterior mode and 90% CrI based on a grid-based posterior scan) were made for ten simulated datasets (sample size n = 1000) and the mean is shown here for each scenario and method. Each plot compares the performance of the two indicated methods as follows: The x-axis indicates the difference in the absolute error of the two methods’ estimates divided (scaled) by the true parameter value (if non-zero)—here absolute error is defined as the magnitude of the estimate minus the true parameter value; the y-axis is the difference in the CrI width of the two methods’ estimates divided (scaled) by the true parameter value (if non-zero). Alongside the legend is the guide to understand the simulation result plots indicating the quadrants where one method outperforms the other. See the legend for simulation result plots in conjunction with Methods and S2 Fig to obtain a complete description of the simulation scenario (including measurement noise setting) underlying each dot in the plots. Note that S2 Fig is an expanded version of the figure shown here containing additional results on KC vs. SC, as well as medium inference difficulty and medium assay sensitivity.

We simulated single- and k-cell data (using k = 10 and 50) subjected to measurement by an assay that would suffer from increasingly missed detection as the input mRNA level is lowered and additional measurement noise under a range of scenarios, and used the resulting single-cell data (SC), k-cell data (KC), or both (SCKC) to infer the value and statistical uncertainty of the CHPs (using a posterior surface scanning procedure; see Methods). The number of samples was fixed at *n* = 1000 across all three approaches–i.e., *n* single-cell, *n* k-cell, or *n*/2 single-cell and *n*/2 k-cell samples were used. Data were simulated under scenarios reflecting low, medium and high levels of difficulty, corresponding to, respectively, high *π* and low *σ* (i.e., high ON fraction and low cell-to-cell variation among ON cells), medium *π* and *σ*, and low *π* and high *σ* (i.e., low ON fraction and high cell-to-cell variation among ON cells) (see S1 Fig and Methods), as well as assays with varying detection efficiencies (bad, medium and good sensitivity assays corresponding to, respectively, 18%, 50% and 82% average detection of single-cell samples and nearly perfect detection of all k-cell samples; see Methods). The simulated data was further subjected to known, realistic sources of experimental noise including sampling, amplification and efficiency noise [12] using five distinct noise configurations (see S1 Fig and Methods). Assessing such diverse scenarios is important and informative since a wide range of possibilities is expected across the tens to thousands of genes targeted by multiplexed techniques such as microfluidic qPCR and RNA-Seq. Performance across the input data types was evaluated via the error (differences between inferred and true values) and statistical uncertainty of the parameter estimates (Fig 2 and S2 and S3 Figs).

Our single-condition simulations revealed that 1) as expected, SC’s performance on inferring both discrete and continuous heterogeneity (*π* and *σ*) was worst in medium- or high-difficulty scenarios using assays suffering from sensitivity issues (Fig 2 and S2 Fig); 2) SCKC was comparable to KC across a range of scenarios, and tended to be better than KC when k is larger using assays of medium to high sensitivity for inferring *π* and *μ*—these were similar to the scenarios under which SC is better than KC when high sensitivity assays are used (see Fig 2 and S2 Fig). Note that SCKC’s inferred values for *π* and *μ* (and to a lesser extent, SC’s) tended to center more tightly around the true values than those inferred from KC, because a given k-cell expression distribution for a larger k could be explained by scenarios involving different combinations of *π* and *μ* (e.g., more ON cells because of higher *π* but lower mean expression *μ*, or alternatively, less ON cells but higher *μ*) and these possibilities could be better disentangled by having single-cell data using a sensitive assay; under this scenario in the case of SCKC, the samples allocated for single-cell measurements provide unique information not well-captured by k-cell data. Depending on the expression-level distribution of the target genes and the assay detection properties, a larger k may sometimes be warranted to improve detection and therefore SCKC could be advantageous in such situations as suggested by these simulation results (see Discussion). Note that these conclusions held largely across the different measurement noise configurations tested except for a few cases as shown in S2 Fig.

A key aim of QVARKS is to enable a quantitative, comparative assessment of heterogeneity parameters of each gene between two biological conditions. Thus we augmented the single-condition assessments with two-condition simulations. We simulated each gene under two conditions with different ON cell fractions (e.g., 80% ON cells in one condition vs. 50% in the other) and subjected them to the same logistic assay and measurement noise settings as in the single-condition scenarios (see Methods). While the relative performance trend of the three methods in inferring parameter differences between the two simulated conditions tends to be similar to that when inferring single-condition parameters (S3 Fig), the performance difference among methods, particularly that between SCKC and KC, was less pronounced in the comparative setting. Again, as above, the measurement noise setting tends to have little effect on the relative performance of different methods except for a few cases as shown in S3 Fig.

Examining single- and two-condition simulation results together revealed differences in how measurement noise affects inference outcomes. As expected, as more noise is added, all three methods suffered from increased error in their estimates within individual conditions because none of the methods explicitly model experimental noise. However, such noise-induced error appeared largely mitigated when comparing across two conditions (S4 Fig), likely because such errors tend to cancel out in comparative analyses between two conditions. However, under some comparative scenarios, such as when both the mean expression and cell-to-cell variation are different between the two conditions, estimates of differences could still be error-prone due to the dependence of technical noise on average/mean expression. This error/bias can potentially be handled by explicitly checking the mean-variance relationship to assess whether the observed difference in heterogeneity of a given gene can be accounted for by changes in the average expression alone between two conditions (as illustrated in the biological application below; see also S11 Fig).

In summary, our simulation results revealed scenarios where different input data types can each offer advantages. We confirm that when assay sensitivity is high, SC can be desirable particularly for providing directly observable estimates of discrete heterogeneity (*π*). As the assay sensitivity lowers and the inference difficulty increases, the advantages of KC become apparent, at the cost of masking biological heterogeneity at larger values of k. Our simulations revealed that SCKC tends to offer the best of both SC and KC under many scenarios, thus suggesting that simultaneous generation and integration of the two data types can be a robust, valuable approach, particularly under multiplexed settings where different genes would fall under different inference difficulty and detection scenarios as simulated above.

### Using simultaneously generated single- and k-cell data to compare the precision of SC, KC and SCKC

Since certain features of real data cannot be fully captured by simulated data, we next sought to assess the relative performance of the three input data modes using single- and 10-cell data (i.e., k = 10) we had jointly generated for studying cell-to-cell expression variation of human macrophages in resting conditions (control, CNT) vs. those exposed to inflammatory cytokines for 24 hours (IFNγ together with TNFα, hereafter referred to as IFNT) (see full description of this data below and in Methods). We performed several analyses to assess relative precision, or conversely statistical uncertainty, using appropriately downsampled data so that the sample sizes were the same across SC, KC and SCKC (see Methods). Here we can only assess precision rather than error (the difference between the true and the estimated values), due to the lack of ground truth about parameter values in real data.

**Fig 3:**
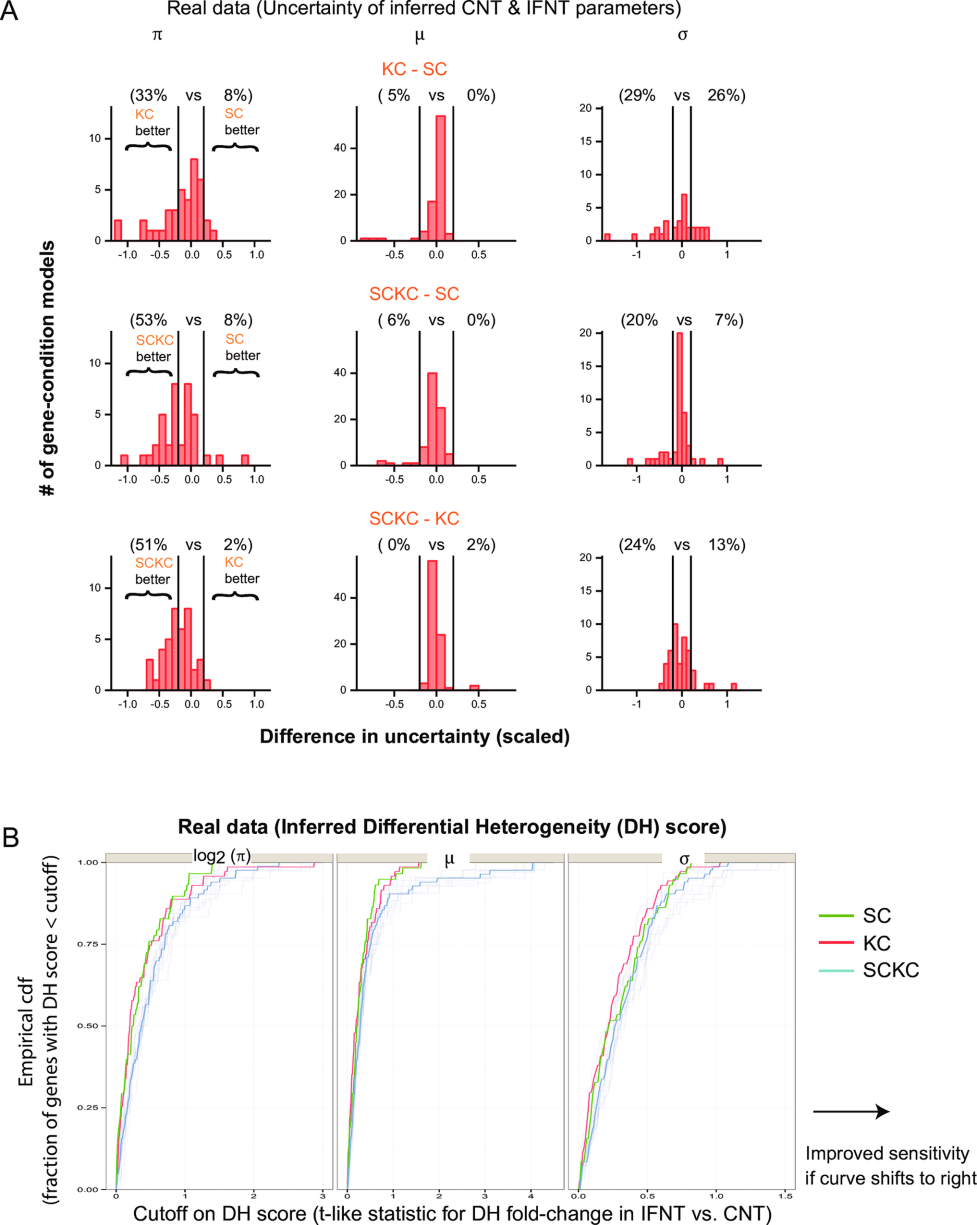
Comparison of SC, KC and SCKC methods using real data. Experimentally generated single- and ten-cell data obtained from resting (CNT) and inflammatory-activated (IFNT) human macrophages are used for assessing the three methods. Note that for these evaluations, ten random downsamplings of the data were performed as described in Methods to make SCKC sample size the same as that of SC or KC. Results from one of these downsamplings chosen at random is shown in (A) given that other downsamplings show similar trends, and results from all 10 downsamplings are shown in (B) as lightly shaded lines along with a darkly shaded blue line connecting the median values across runs. **(A)** Comparing the precision (or uncertainty) of the parameter estimate of the indicated methods. For gene-condition combinations (GCCs) whose parameter estimates are similar between the compared methods (see Methods and S5 Fig), the difference in CrI width (reflecting uncertainty/precision) between the two compared methods scaled by the average parameter estimate is computed and shown as a histogram. Atop the histogram are the percent of GCCs having at least a 20% precision advantage in one method vs. the other in the indicated comparison (see Methods). **(B)** Differential heterogeneity (DH) comparisons. The empirical cumulative distribution function (CDF) of a t-like fold-change statistic for assessing differences between IFNT vs. CNT in the indicated parameter is shown. The statistic corresponds to the difference between the average estimate in the two conditions divided by the average uncertainty (CrI width). The indicated CDF fraction (y axis) is out of all successfully modeled genes, which is 71 for KC, 58 for SC and 41.5 (median) across different SCKC runs.

We first assessed the precision of estimated parameters as quantified by the credible interval (CrI) width reflecting the amount of statistical uncertainty about the true value of the parameter. In particular, we focused on genes with similar inferred values across the three input modes to avoid confounding from potential correlation between precision and error/bias. This analysis revealed that SCKC tends to provide more precise estimates for *π* and *σ* than either SC or KC alone across a larger fraction of genes (Fig 3A and S5 Fig). By using a t-like fold-change statistic to assess statistical power for detecting changes in heterogeneity (DH) between two conditions, this improved precision of SCKC in comparison to SC or KC also translated to mild increases in sensitivity for detecting DH, especially for the *π* and *μ* parameters (Fig 3B).

**Fig 4:**
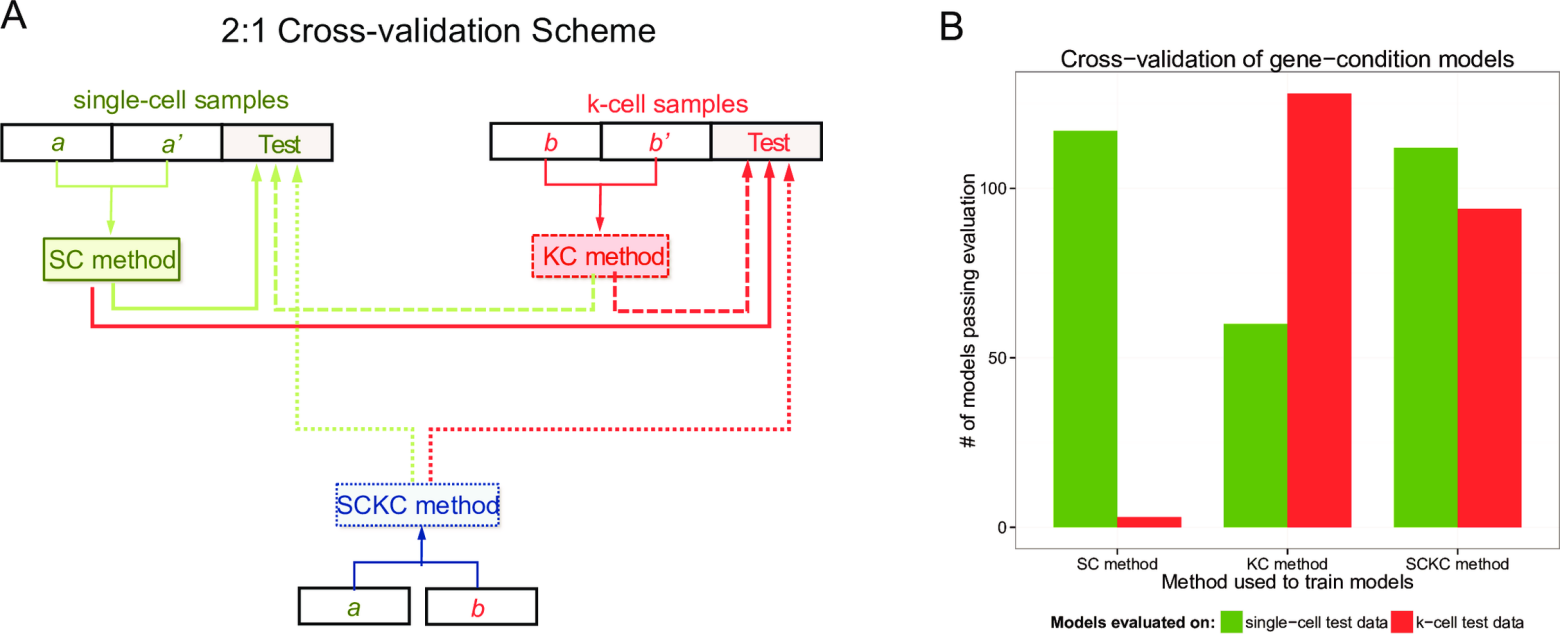
Cross-validation of SC, KC and SCKC methods using real data. **(A)** Illustration of the 2:1 cross-validation scheme. Note that the SCKC method’s input data is of the same sample size as the SC or KC method. **(B)** Bar plots showing the number of GCC models trained using 2/3^rd^ of the data via the indicated method (SC, KC or SCKC) that successfully fit the unseen 1/3^rd^ of the data. Here the fit is evaluated based on applying our AD-test model assessment criteria on either single-cell (green bars) or k-cell (red bars) test data.

We next evaluated whether using single-cell data alone would lead to inferred parameters consistent with k-cell data and vice versa. In a perfect world where the single-cell assays have 100% detection and the value of k is sufficiently small so that masking of biological cell-to-cell heterogeneity due to convolution of k cells is minimal, single- and k-cell data would reveal similar information about cell-to-cell heterogeneity. In reality, these two data types may provide different parameter estimates due to a variety of reasons, including their differences in susceptibility to detection limit problems, and thus it would be informative using simultaneously generated single- and k-cell data to formally assess their consistency. Towards this end, we performed a cross-validation analysis by randomly dividing our data (84 single- and randomly down-sampled 84 ten-cell samples per condition (IFNT and CNT)) in 2:1 ratio into training and testing sets (Fig 4A). We used the training data to infer models separately using SC or KC and the testing fraction to assess how well the inferred model fits using our AD-test model assessment criteria (see Fig 1B and Methods). This analysis showed SC-inferred models tend to explain only the corresponding single-cell test data well but not the k-cell test data, and similarly but to a lesser degree, the KC-inferred models are more aligned with the k- than single-cell test data (Fig 4B). However when both training data types were used (SCKC), the inferred models fit both the single and k-cell test data substantially better than using the SC or KC inferred models alone (Fig 4B). To an extent, these observations are to be expected given that SCKC uses both single- and k-cell training data (half of each to keep the same sample size across methods). Yet, importantly, our results revealed that single- or k-cell analysis on their own could lead to models irreconcilable with the other data type, each of which contains important information about the expression distribution of a gene across single cells. Given the highly multiplexed nature of modern gene expression assays, a sizable number of genes could fall under such “incompatible” scenarios, thus providing support for the generation, cross-checking and proper integration of both data types when possible.

### Using bulk RNA-Seq data and differential expression (DE) performance to assess QVARKS

While the focus of QVARKS is to infer CHPs within a single condition or compare CHPs between two conditions, several existing single-cell approaches focus on assessing differential expression between conditions using single-cell data. QVARKS can also be used to assess DE using one of the three possible input data modes. Assessing DE affords us another way to check our model assumptions and the overall method because QVARKS computes DE using the posterior distribution of its model parameters and thus achieving reasonable DE results require that our inferred posterior distributions be accurate. Here following previous studies [11,18], we view the DE estimates (log_2_-fold-change (log_2_(FC)) of the average expression of a gene between two conditions) computed from bulk, cell population-level RNA-Seq data as “ground truths”. Our bulk RNA-Seq data was generated in a related study in the same macrophage conditions as our single- and k-cell data. In addition to comparing against bulk RNA-seq, we also assessed QVARKS’ performance in recapitulating bulk DE relative to that of two other single-cell based DE methods: MAST [18] and SCDE [11] (S6 Fig). Specifically, we applied MAST and SCDE to our macrophage qPCR data and compared with QVARKS running in SC, KC or SCKC modes using relative (Pearson correlation shown in S6A and S6B Fig) and absolute (average squared error shown in S6C and S6D Fig) measures of recovering ground-truth fold-change values. Evaluation based on the relative, correlation based measure revealed that QVARKS SCKC and KC nicely capture bulk level DE and perform comparably to MAST and SCDE, and QVARKS SC performs comparably once genes with large CrI are removed (S6A Fig). QVARKS SC in general yields more parameter estimates with large uncertainty (large CrI) as has also been observed above (Fig 3A), thus removal of these genes led to improved performance. Evaluation based on the absolute measure showed that DE estimates from all QVARKS input modes were well-calibrated with respect to the ground-truth values, and thus all had relatively low absolute error (S6C Fig). Thus, the overall DE performance of QVARKS suggests that our model assumptions and inference procedures are reasonable and capture the data well.

### QVARKS reveals condition-dependent CEV in resting vs. inflammatory human macrophages

To assess and illustrate the practical applicability and utility of QVARKS, we next applied QVARKS to the full single/10-cell macrophage data set introduced above. Here we focused our analysis using SCKC as the input data so that we can use all available data; SCKC also tends to provide the most robust results as suggested by the evaluations above. The data was obtained in a related study using fluorescence activated cell sorting (FACS) of single and ten cells followed by microfluidic qPCR (measuring 93 transcripts and 3 spike-in control RNAs; see Methods). Macrophages are immune cells that exhibit diverse environment-dependent phenotypes and hence are good models to study how the environment shapes CEV in transcript levels, such as changes in the fraction of ON cells (*π*). Environment- or signal-induced changes in gene expression have largely been assessed by measuring alterations in average expression using a population of cells, but measuring the average alone could miss changes in CEV. By taking advantage of the posterior distributions containing measures of statistical uncertainty around the inferred heterogeneity parameters, we can begin to quantitatively assess changes in CEV in resting vs. IFNT-activated (inflammatory) macrophages.

Our Bayesian procedure successfully modeled and obtained posterior distributions of model parameters for 53 and 60 genes in CNT and IFNT, respectively. Of these, 41 are shared between CNT and IFNT, and hence their CEV can be quantitatively compared across these two conditions (see below). A majority of the remaining genes failed model assessment due to scarcity of detected cells (observed ON cells less than 5 in single-cell data for 23 failed genes in CNT and 17 failed genes in IFNT). Since these genes are inherently difficult to work with for inferring CHPs (e.g. inferring ON-cell variance from 5 or fewer cells is challenging), they could essentially be filtered out *a priori*. Under such a filter, the model assessment rate increases to 71–75% (47 out of 66 filtered genes in CNT, and 51 out of 68 in IFNT were successfully modeled). Thus, consistent with our DE assessment above, our model assessment indicates that for many genes, the data is captured well by our models, suggesting that our model assumptions, including distribution choices, are also robustly supported by the data.

**Fig 5:**
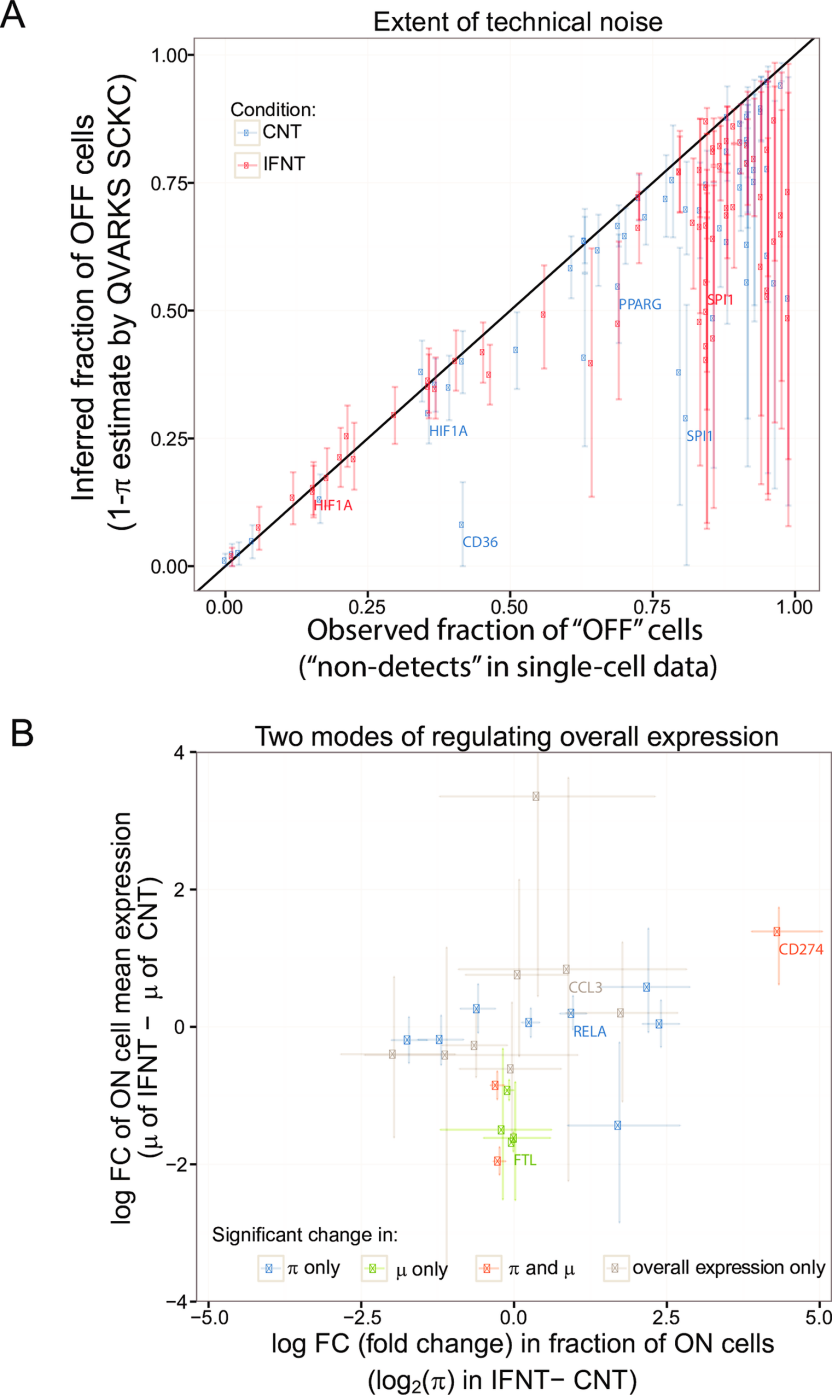
Application of QVARKS SCKC reveals technical drop-offs and basis of bulk differential expression. **(A)** Extent of technical noise. Comparing the inferred (y) vs. observed (x) fraction of OFF cells across genes shows that genes with higher fractions of OFF cells were affected the most by imperfect detection. Only genes successfully modeled in either the IFNT or CNT conditions are shown; the 90% credible interval (Crl) is indicated by the lightly shaded line around the mean of the posterior distribution. Several example genes associated with inflammatory or anti-inflammatory processes in macrophages are marked. **(B)** Distinct modes of regulating differential expression. The 23 differentially expressed genes based on average expression in the cell population in resting vs. IFNT conditions are shown in a scatter plot depicting differences in the fraction of ON cells (x) against differences in the mean of ON cells (y). The CHPs inferred using QVARKS are used to classify these genes into those with significant (adjP < 0.05) changes in: 1) only the fraction of ON cells, 2) only the mean expression of ON cells, 3) both, or 4) neither. The 90% Crl is indicated by the lightly shaded lines around the mean of the posterior distribution as before.

Despite the relatively high sensitivity of qPCR assays, signs of imperfect detection were apparent given that the average expression derived from single-cell data tended to be consistently lower than that obtained from 10-cell data (divided by 10) for transcripts expressed at medium or low levels (S7 Fig). Thus, some apparently “OFF” cells likely had some non-zero level of expression that simply escaped detection. Indeed, the *inferred* fraction of OFF cells (1-*π*) for a majority of genes in both conditions was consistently lower, albeit only slightly in most cases, than that observed on single-cell data alone (Fig 5A). As expected, genes with a higher observed fraction of OFF cells also tended to have larger uncertainty for *π* because it is more difficult to narrow down model parameters (including ones associated with detection) using information from just a few ON cells (analogous to the “high difficulty” simulation scenario; see Fig 2). However, even for such “difficult” genes, not all of the OFF cells can be attributed to escaped detection–the inferred fraction of OFF cells is at least 25% for a majority of these genes even after accounting for statistical uncertainty (based on counting genes whose CrI is above 0.25 in Fig 5A). Thus, our Bayesian analysis integrating single- and 10-cell data helped estimate the proportion of OFF cells attributable to detection issues and thereby helped obtain better estimates of the expression distribution among single cells for a majority of the genes we measured.

Bulk differential expression (DE) between two conditions can be attributed to a combination of changes in the fraction of ON cells and in the mean expression level among ON cells [7]. Here QVARKS provides a rigorous framework to explore the relationship between bulk differential expression (DE) and alterations in heterogeneity parameters *π* and *μ* [7] upon inflammatory activation. We assessed the status of these parameters for 23 DE genes between IFNT and CNT conditions (adjusted P-value or adjP < 0.05) determined using the posterior distribution of the average mRNA level (reflected by the mean *μ* of ON-cells weighted by *π*; see Methods)–these genes were largely similar to DE genes obtained directly from 10-cell data (see Methods and S8 Fig). Eight of the DE genes had significant changes in *π* but not *μ* (e.g., *RELA*), while four had the opposite behavior (e.g., *FTL*), and the rest either had significant changes in both (e.g., *CD274*) or neither (Fig 5B). Note that many genes in the last category lacked significant change in *π* and *μ* not necessarily because the mean fold changes are small, but more because the statistical uncertainty around their inferred fold changes is large. Taken together, our results show that in macrophages adapting to inflammatory stimulation, both “digital” (altering *π*) and “fine-tuning” (altering *μ*) modes of regulating gene expression are prevalent.

**Fig 6:**
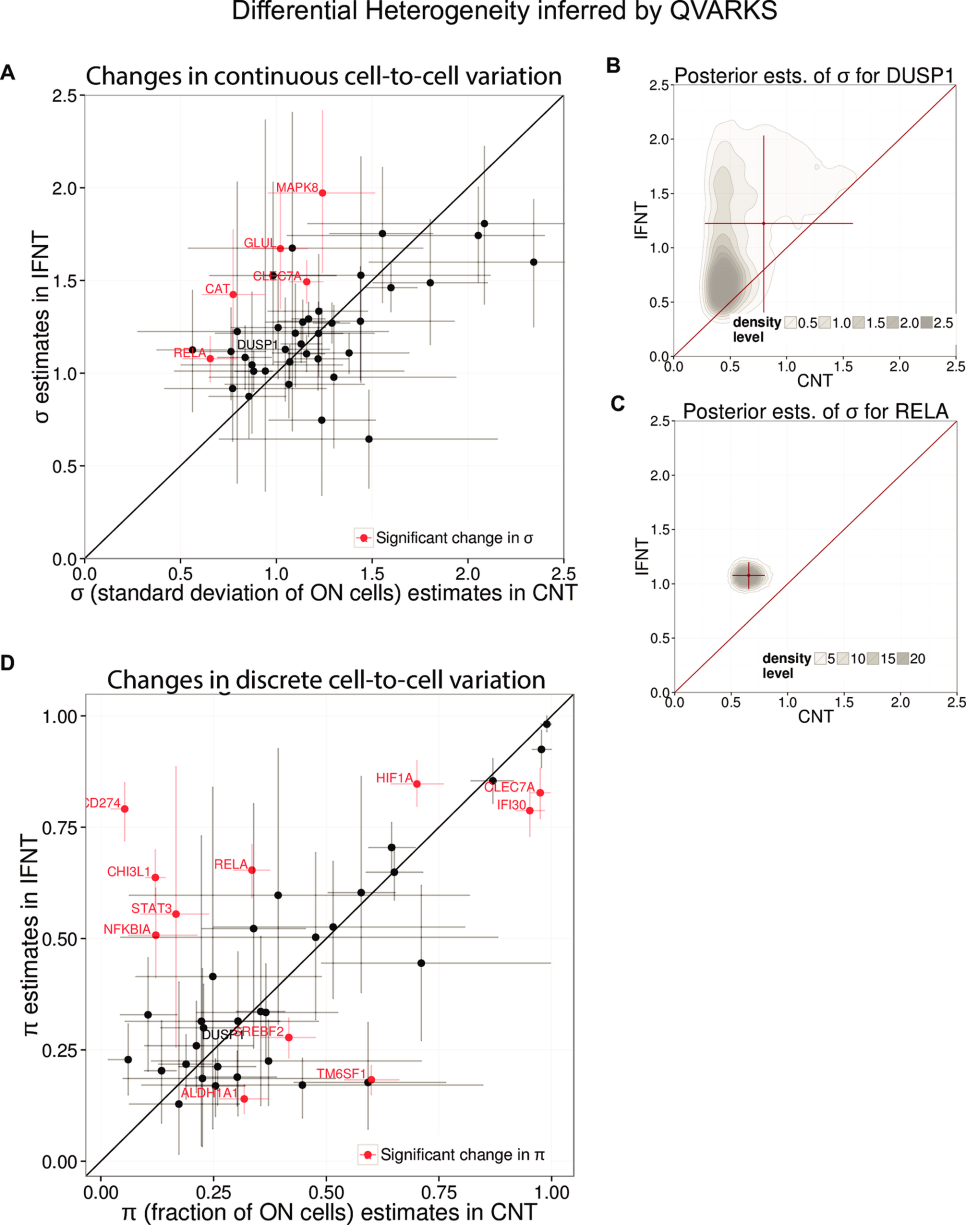
Application of QVARKS SCKC to assess differential heterogeneity. QVARKS was applied to assess significant (adjP < 0.05) changes in *π* and *σ* using joint single- and 10-cell data obtained from human macrophages in resting (CNT) vs. IFNT-activated conditions. **(A,D)** Continuous heterogeneity as reflected by *σ* (A) or discrete heterogeneity as reflected by *π* (D) were significantly altered in IFNT (y) relative to CNT (x) for some (indicated in red) of the 41 successfully modeled genes. The 90% Crl is indicated by the lightly shaded lines around the mean of the posterior distribution. **(B,C)** The posterior distribution of *σ* for *DUSP1* (B) and *RELA* (C) are shown to illustrate why only *RELA* was deemed significantly changed between the two conditions. The 90% CrI around the posterior mean shown in (A) is indicated here as red lines.

A key application of QVARKS is to assess changes in heterogeneity parameters (or DH), such as asking whether environment shapes cellular heterogeneity. Thus, we next inferred differences between IFNT and CNT in terms of 1) the standard deviation of expression in ON cells (*σ*) reflecting continuous heterogeneity and 2) the ON-fraction (*π*) reflecting discrete heterogeneity (Fig 6). The inferred differences in these CHPs for successfully modeled genes were also verified to be robust against well-to-well variation in detection efficiency quantitated using spiked-in ERCC control mRNAs (see S9 Fig; note that detection efficiency was a major source of technical noise assessed in [12]). In addition to *π* and *σ*, comparative assessment of heterogeneity across conditions can also be extended to other notions of heterogeneity, such as the Shannon entropy function computed using *π*, by taking advantage of our Bayesian approach’s ability to handle inference of any function of the CHPs (see S10 Fig and Methods).

Our analyses revealed both shared and condition-specific patterns of CEV (Fig 6A and 6D). Genes such as *FTL* had similar continuous and discrete heterogeneity across conditions. However, *RELA*, a component of the NFκB complex, for example, had a higher fraction of ON cells (adjP = 2.15e-05, calculated as described in Methods) as well as higher variability among ON cells in IFNT than CNT (adjP = 0.012, Fig 6C). Such changes in continuous variability could not be solely attributed to differences in average expression among ON cells (*μ*) or fraction of ON cells (*π*) between the conditions (S11 Fig). The inferred change in *σ* across the two conditions for genes such as *RELA* was also robust against sampling noise when random (50%) downsamplings of the data were assessed (S12 Fig), and is thus potentially reflective of changes in biological cell-to-cell variation. Since NFκB is a key transcription factor mediating responses to diverse inflammatory signals in macrophages [25], an increase in the heterogeneity of *RELA* expression upon inflammatory activation by IFNT suggests that elevating the response diversity among cells in, for example, infected tissues may play an important functional role, such as to counteract bacterial targeting of activated cells or to prevent detrimental over-inflammation. *CLEC7A* also showed changes in both types of heterogeneity, albeit in opposite directions, i.e., up in continuous (adjP = 0.002) and down in discrete (adjP = 0.0006) variations. Differences in *σ* could, for instance, reflect changes in stochastic dynamics during transcription and mRNA degradation, or in negative feedback regulation. Thus, changes in both heterogeneity parameters in inflammatory vs. resting macrophages can be robustly detected by our approach. It will be interesting in the future to study the underlying mechanisms and function of such distinct gene regulation modes revealed by the differential heterogeneity and differential expression tests enabled by QVARKS.

## Discussion

Environment- or signal-induced changes in gene expression have largely been assessed by measuring alterations in average expression using a population of cells. However, measuring the average alone could miss changes in cell-to-cell heterogeneity. Assessing such changes in a statistically rigorous manner has been challenging, in part due to difficulties in disentangling technical vs. biological variations in single-cell measurements. To help overcome these challenges, we have developed a flexible computational method for inferring CHPs within a cell population or across two cell populations using single-cell, k-cell, or both data types simultaneously, by obtaining measures of statistical uncertainty around the inferred heterogeneity parameters so that the significance of an observed change can be evaluated. Here using inflammatory activation of human macrophages as a first biological application, we have uncovered a number of significant changes in the fraction (*π*) of and/or variance (*σ*) among ON cells. Furthermore, we have shown that changing *π* alone (“digital” gene expression) can contribute to changes in mean expression at the bulk level. Our approach can also be used to explore whether cellular heterogeneity can change without accompanying changes in average expression, e.g., increasing *π* and *σ* while reducing *μ* such that the population average is unaltered. Indeed, it is increasingly recognized that cell-to-cell variations could themselves be regulated both genetically and environmentally without affecting the average expression level to, for example, reduce the chance of noise-induced cellular activation by lowering heterogeneity or hedge against environmental fluctuations by ensuring sufficient variation across cells in a population [17]. However, it is worth noting that despite our modeling of technical factors such as misdetection, interpreting continuous CEV parameters (the variation among ON cells, or *σ*) can be more challenging in general than interpreting discrete CEV parameters (the fraction of ON cells, or *π*). For example, other sources of technical noise are still possible and thus the biological and technical sources may not be fully disentangled, and additional checks, such as those in S11 Fig based on linear regression, do not account for non-linear relationship between mean and variance, and could violate some linear regression assumptions (such as error-free measurement of independent variables and homoscedasticity). One way to help disentangle biological from technical variation in the future is to utilize dilution-series experiments to obtain an estimate of the technical variation of all gene assays at different average expression levels, although sampling/pipetting noise also need to be considered properly at lower ends of the concentration scale.

The unique ability of QVARKS in handling the three input data types (SC, KC and SCKC) allowed us to assess their relative performance for the first time under a common Bayesian analysis framework. While analyses of both simulated and real data suggest that there are situations under which each of single- or k-cell input data types can offer advantages, in general combining single- and k-cell data—when both are generated simultaneously—could yield more robust estimates of CHPs compared to using single- or k-cell data alone across a range of scenarios, particularly for genes with moderate to poor detection. A further practical appeal of using single- and k-cell data jointly, particularly in multiplexed settings such as microfluidic qPCR and RNA-Seq where tens to thousands of genes are measured, is that the single-cell data for genes with good detection (e.g., highly expressed genes) can be directly analyzed to enable applications that require measurements of multiple genes within individual cells, such as identifying novel cell subsets. However, our approach is flexible and therefore can also be applied to single-cell data for assessing CHPs within a single condition or comparatively between two conditions.

Our methodological framework can be extended to incorporate additional parameters or use alternative model parameterizations to account for features such as multimodality within ON cells, e.g., in samples comprising mixed cell types or subsets. But the inference accuracy would be constrained by sample size. For example, each additional mode would require three extra parameters per biological condition in order to capture its frequency, mean and variance (assuming using a log-normal distribution). Thus, it would only become feasible to learn the extra parameters for such models when larger sample sizes are available, which will likely be the case in the near future as the experimental cost of profiling continues to drop with the introduction of new technologies [5]. Here we chose to use the unimodal distribution for ON cells for several reasons. First, it is simple, yet captures our single- and k-cell data well—as discussed above, our model assessment criteria indicated that this parameterization was sufficient to successfully fit a majority of genes. Among the remaining genes, the predominant reason for the lack of fit appeared to be the scarcity of ON cells rather than bimodality among them: there are only two GCCs with more than 10% ON cells that appeared to be multi-modal (TIGD6 in CNT and PTGS1 in IFNT according to Hartigan’s Dip test for unimodality using a relaxed P < = 0.2 cutoff). While additional multimodal genes may be present in our study, they were not evident at our current sample size.

The model assessment procedure used in this study, the default in the R package implementing our QVARKS approach, leans on the stringent/conservative side (i.e., allows less genes to pass model assessment), in that we required most data sets generated from each of the posterior parameter draws to be statistically indistinguishable from the observed data (see Methods). Users of the R package can choose other forms of model assessment, including a variant of the one used here where data simulated from different posterior parameter draws are concatenated into one dataset, and a single AD test is carried out to assess the concordance of this dataset against the observed data. This approach can work well for genes with just a few ON cells since concatenating all simulated datasets into one provides a larger sample size to enable a more robust comparison against observed data. However, one advantage of the default approach is that it matches the sample sizes of the simulated and observed data when performing each of the multiple AD tests. Another model assessment criterion that our R package supports is based on the well-accepted posterior predictive p values (ppp; [23,24]), wherein we empirically test if a particular aspect of the observed data, such as mean or variance of observed ON cells, is captured well by simulated datasets generated from the posterior parameter draws. However, the ppp requires the user to choose which aspect of the data (e.g., mean and/or variance) to use, and does not use information from the whole data distribution the way the AD test does. The vignette/manual of the QVARKS R package illustrates and compares these model assessment options. Having multiple model assessment options allows the users to have more means to determine whether to trust a particular gene’s fitted model, and depending on the goals of the analysis, proper tradeoffs can be made between ensuring that model assumptions are comprehensively met vs. discarding expensive single- or k-cell data. For example, consider a gene that is expressed in a high number of ON cells in condition A but in very few in condition B. If the goal were to compare the continuous heterogeneity of genes between the two conditions, it would be challenging to analyze this gene as the very few ON cells in condition B would yield unreliable estimates for *σ* in that condition. However, changes in the discrete heterogeneity (*π*) or average differential expression can be more reliably studied for this gene between the two conditions.

When the k-cell approach is used, the optimal value of k could depend on the cell type and assayed genes, thus empirical assessment is needed for choosing k. For example, a dilution series of bulk mRNA can be profiled to estimate the minimum value of k that would provide reasonable detection for most genes. Our simulation assessment suggests a tradeoff between the value of k and sensitivity: increasing k would better mitigate detection issues at the cost of masking biologically relevant heterogeneity from convolving single-cell samples. Indeed, in general k should be large enough to avoid detection issues, but sufficiently small to avoid such masking effects where the relative contribution of biological cell-to-cell variations would become increasingly small and thus undetectable due to technical noise. Similarly, the optimal proportion of single- vs. k-cell samples to profile is context-dependent and best chosen using an empirical approach. When no prior information is available, as in this study, one could start with an equal proportion of both data types (e.g., 100 single- and 100 k-cell samples). One can then use this data to estimate the relative contributions of each data type to the model likelihoods and use this information to help determine the relative proportion of single- and k-cell samples to profile in the next round of experiments. This process can be iterated to fine-tune the proportion until a fixed profiling budget is reached. However, until the cost and time of sample preparation and sequencing drop to negligibly low levels, this iterative strategy can be prohibitive; thus, in practice, it is best to simply profile an equal number of single- and k-cells and only generate more data of one of the data types if it is quantitatively clear that additional data would help with answering the biological question of interest.

The vignette document distributed along with our QVARKS R package describes the functionality of our package using actual data examples and discusses issues related to experimental design (e.g., the dilution-series experiments discussed above when k-cell data is desired) as well as statistical considerations (e.g., data quality assessment, preprocessing, transformation steps, and model assessment) and computational considerations (e.g., scalability of the inference procedure to process a large number of genes via parallelization). Though QVARKS was primarily developed to analyze single-cell qPCR datasets, as a proof of concept, the vignette also illustrates how QVARKS can be applied to an externally pre-processed single-cell RNA-Seq dataset using a parallel computing cluster. Our approach thus provides a principled and practical method to explore cellular heterogeneity in diverse settings.

## Methods

### Ethics statement

Peripheral blood was obtained by leukapheresis from a de-identified healthy donor by the NIH Blood Bank, Department of Transfusion Medicine, National Institutes of Health Clinical Center under IRB-approved protocol 99-CC-0168.

### Model description and likelihood function

We motivated and described the parametric model of gene expression in single- and k-cell samples from a cell population in the main text. Here, we formally specify the model along with the likelihood function of model parameters. As shown below, the choices of specific distributions in our model are inspired by earlier single-cell modeling studies (eg. bimodal mixtures of ON/OFF cells and log-normally distributed ON-cell transcript levels assayed using qPCR [7] or RNA-Seq [18,21], and logistic detection behavior of qPCR [8,22] or RNA-Seq [11] assays). We extend the existing single-cell models by integrating both single- and k-cell data, explicitly modeling imperfect detection assays and handling more than one environmental condition under which a gene is profiled. Multiple environmental conditions are handled by keeping the detection behavior parameters the same across conditions (which is reasonable as the same gene assay is used across conditions) and letting the CHPs (π, μ and σ) be condition-specific. We present the single-condition model first and then point out what changes need to be made to handle the two conditions focused in this study. An interesting future direction would be to extend these single/two-condition models to handle more complex experimental designs, e.g., those involving more than two conditions and handling covariates such as donor information, batch effects, and cell size.

We extend a model of “underlying” or “true” single-cell expression in an earlier study [7] to obtain a model of “measured” single-cell expression that handles imperfect detection as follows. Let G denote the expression of a gene transcript (i.e., counts of transcript copies without any log transformation) in a random cell in the population. Then the bimodal mixture model for G could be written in random variable notation as G = I X, where I ∼ Bernoulli(π) is an indicator variable that is 1 for an ON-cell of the gene with probability *π* and 0 otherwise, and *log*_2_*X* ∼ *Normal*(*μ*, *σ*). Note that the log of average overall expression is given by log_2_(*E*(*G*)) = *log*_2_(*E*(*I*)*E*(*X*)) = log_2_ *π* + *μ* + *σ*^2^ log_*e*_(2)/2 (in line with the intuition that both fraction and mean expression of ON cells determines the overall expression output, with *σ*^2^ contribution coming from cells in the tail of a log-normal distribution). The measured expression *H* (e.g., 2^*Et*^ values of the gene across single-cell samples in a given condition, where Et = 40-Ct is a unit of measurement for qPCR assays as described below) is either detected at the true underlying measurement *G* with a certain detection probability *DP*(*G*) (which ranges from 0 to 1 and increases with expression strength in a logistic manner) or not detected (2^*Et*^ = 0) otherwise. So *H* = *J*(*G*) *G*, where *J*(*G*) ∼ *Bernoulli*(*DP*(*G*)) is an indicator variable that is 1 for detected measurements and 0 for non-detects, and *DP*(*G*) is a logistic function with intercept *c* and slope *m*, i.e., ${DP}_{c,m}(G)=1/(1+\exp\left(-\frac{{log}_{2}(G)-c}{m}\right))$. This parameterization reflects the standard way of modeling the probability of a binary response, such as non-detect vs. detects in expression profiles. We can now write the likelihood function of the model parameters given single-cell expression measurements *h*_1_,*h*_2_,…,*h*_*n*_ of the gene in *n* cells for a given condition as below. Here, *P*_*μ*,*σ*_(*X* = *x*) denotes the log-normal probability density function evaluated at *x* or equivalently the normal density function with mean *μ* and variance *σ*^2^ evaluated at *log*_2_(*x*).

$$\begin{array}{l} L(\pi ,\mu ,\sigma ,c,m\;|\;h_{1},h_{2},\ldots ,h_{n})=\prod_{i=1}^{n}L(\pi ,\mu ,\sigma ,c,m\;|\;h_{i}),\;where\\ L(\pi ,\mu ,\sigma ,c,m\;|\;h)=\pi \;P_{\mu ,\sigma }(X=h)\;{DP}_{c,m}(h)\;if\;h>0,\;and\\ \;=(1-\pi )+\pi \;\int_{g=0}^{\infty }P_{\mu ,\sigma }(X=g)\;{(1-DP}_{c,m}(g))\;dg\;if\;h=0. \end{array}$$

Since each k-cell measurement is modeled as aggregating mRNAs from k randomly chosen independent cells and subjecting it to the same imperfect detection as single-cell data, we can write “true” k-cell expression $V=\sum_{i=1}^{k}G_{i}$ (with the *G*_*i*_s being independent and identically distributed as *G* above, and hence fully defined by the single-cell expression parameters π, μ and σ) and the k-cell measurement *W* = *J*(*V*) *V* (in the same way that *H* is obtained from *G* using the *c*,*m*-parameter logistic “assay”). Since the true single-cell distribution *G* is bimodal, the true k-cell distribution *V* is multimodal depending on how many ON cells constitute a particular k-cell pool (denoted l). The likelihood function of this multimodal distribution can be approximated either empirically or analytically [15]:

1. The empirical (or Monte Carlo) approximation is straightforward and can work seamlessly for any choice of single-cell distribution, probabilistic detection models and k value, but it is more computationally intensive. This approach involves building kernel density estimate (KDE; using R’s density function with default values for instance) from repeatedly drawn k-cell measurements, where each repeat involves summing k independent samples from the underlying single-cell distribution and simulating their detection by the logistic “assay”.
2. The analytical approximation is more computationally efficient—it involves marginalizing out the unknown value of l and approximating the distribution of a sum of l ON-cell transcript counts for each l (denoted $X_{\left(l\right)}^{}=\sum_{i=1}^{l}X_{i}$, with *X*_*i*_s being independent and log-normally distributed as *X* above) by another mean/variance-matched log-normal distribution as done in an earlier study [15].

The empirical approximation becomes more accurate by increasing the number of repeated samples but at the burden of increased computational time, and the log-normal sum approximation we use, called the Fenton-Wilkinson approximation, is accurate when the standard deviation *σ* is not too large [26]. Both approaches yielded similar likelihood values for the parameter configurations we checked (involving *σ* values ranging from 0.25 to 2, and using 10,000 samples to build each KDE)–so we eventually chose the analytical approach in this study for computational efficiency reasons. The likelihood function of the model parameters given k-cell measurements *w*_1_,*w*_2_,…,*w*_*n*_ of a gene in *n* k-cell pools for a given condition can either be directly read off from the KDE for each measurement and multiplied together in the empirical approach, or given by the formula below in the analytical approach. Here, *P*_*μ*,*σ*_(*X*_(*l*)_ = *x*) denotes the Fenton-Wilkinson approximated probability density function of the sum of l ON-cell transcripts evaluated at *x*, and Pπ(#ONcells=l)=(kl)πl(1−π)k−l denotes the probability of observing l ON-cells in a random k-cell pool.

$$\begin{array}{l} L(\pi ,\mu ,\sigma ,c,m\;|\;w_{1}^{},w_{2}^{},\ldots ,w_{n}^{})=\prod_{i=1}^{n}L(\pi ,\mu ,\sigma ,c,m|w_{i}^{}),\;where\\ L(\pi ,\mu ,\sigma ,c,m\;|\;w)=\sum_{l=1}^{k}P_{\pi }(\#\;ON\;cells=l)\;P_{\mu ,\sigma }(X_{\left(l\right)}=w)\;{DP}_{c,m}(w)\;if\;w>0\\ \;={\left(1-\pi \right)}^{k}+\sum_{l=1}^{k}P_{\pi }(\#\;ON\;cells=l)\;\int_{v=0}^{\infty }P_{\mu ,\sigma }(X_{\left(l\right)}^{}=v){(1-DP}_{c,m}(v))\;dv\;if\;w=0. \end{array}$$

This likelihood function agrees with intuition that pools of small number of cells retain information on single-cell variations, but that of large number of cells lead to “bulk or masked-out” effect that makes the *π* and *μ* parameters inseparable, as also noted in the main text when presenting simulation results. Because as *k* increases, the binomially distributed number of ON cells in a random k-cell pool becomes more tightly concentrated around the value *kπ*, and so the k-cell mixture asymptotically collapses from (*k* + 1) components corresponding to different numbers of ON cells to a single component whose distribution is the sum of *kπ* i.i.d. log-normal variables. Note that the sum of *kπ* i.i.d. log-normal variables can be (Fenton-Wilkinson) approximated by another log-normal with log-space parameters *μ*’ = ln(*kπe*^*μ*^) + (*σ*^2^ − *σ*’^2^)/2 and $\sigma {’}^{2}=ln((e^{\sigma^{2}}–1)/(k\pi )+1)$. Such a k-cell mixture essentially makes the three heterogeneity parameters inseparable (or non-identifiable) using k-cell data alone, since the same k-cell distribution (given by the same *μ*’, *σ*’) can be explained equally well by different combinations of *π*, *μ* and *σ*. Note that though in reality the k-cell mixture model collapses to more than a single component for large k values, the log-space parameters of these different components would be so close to each other that it would be difficult to separate them out using reasonable sample sizes.

To integrate single/k-cell data and obtain overall log likelihood of the five model parameters given both single/k-cell data, we simply add the log of the k-cell likelihood and log of the single-cell likelihood specified above. To handle two environmental conditions, we add three new parameters *π*″,*μ*″ and *σ*″ for the new condition and use the same likelihood function specified above to compute the likelihood of all eight model parameters given each condition’s single/k-cell measurements separately and finally add the log-likelihood corresponding to all measurements together. Since we use a non-informative prior, the joint posterior distribution is proportional to the likelihood function. In case of an informative prior, the posterior is proportional to the product of the prior probability and the likelihood function (i.e., *P*(*parameters* | *data*) ∝ *P*(*parameters*) *P*(*data* | *parameters*)). In other words, what you know about the parameters after the data arrive is what you knew before, and what the data told you [27].

### Model inference using MCMC-based Bayesian approach

To infer the joint posterior distribution of all parameters, we use a random walk Metropolis (RWM) Markov Chain Monte Carlo (MCMC) procedure with an adaptive tuning phase [27]. The proposal distribution or proposed jump for a RWM MCMC is an independent Gaussian variable in each direction (each of the five parameters for single-condition or eight parameters for two-condition gene expression) with mean 0 and a certain fixed proposal variance. This would work for a posterior with unbounded support, but would be too computationally inefficient for our posterior with bounded support (due to MCMC steps being wasted in non-permissible values of the parameter, which do not satisfy parameter constraints such as 0 ≤ π ≤ 1 and σ ≥ 0). Therefore we use a truncated Gaussian proposal for each parameter, with the truncation ensuring that each new proposed state of the MCMC is always within the constrained parameter range. Note that the pdf (probability density function) of a Gaussian distribution truncated to the interval [*a*,*b*] is 0 outside this interval, and same as the normal Gaussian pdf otherwise, except for a uniform scaling of pdf values within the interval by a constant so that the integral is unity. In the case of qPCR expression data in Et = 40-Ct units [7] (also see below for our qPCR data description), we imposed additional constraints to further improve computational efficiency including 0 ≤ μ, σ, c ≤ 40 and 0 ≤ m ≤ 5. These constraints can also work for other data types such as RNA-Seq data, once the RNA-Seq-based read counts have underwent additional pre-processing steps (such as expression noise thresholding and log-transformation [18,21]) that are required to reveal the bimodal mixture of OFF/ON cells and log-normal distribution of ON-cells for conforming genes. Our implementation is in the R statistical environment and extends the RWM MCMC implementation in the R package *mcmc* that works for any unbounded continuous distribution on R^d^ to the case of a continuous distribution with bounded support (which in our case is the posterior or likelihood function for non-informative priors).

The whole MCMC-based inference procedure involves three phases: a) an adaptive phase where the proposal variance is tuned to achieve good mixing–we use a noisy gradient algorithm as implemented in the R package *JAGS* for this phase using a maximum of 400,000 iterations, b) a burn-in phase where the tuned proposal variance is fixed and the chain is allowed to mix for 20,000 iterations and all resulting samples discarded, and c) a final sampling phase where the actual samples are collected over 200,000 iterations. These iteration counts for single-condition inference are multiplied by two for a two-condition inference, and were determined based on pilot MCMC runs and inspection of convergence diagnostics. Convergence diagnostics such as MCMC trace plots and autocorrelation times are also reported alongside each gene’s inference, and could be used to filter out genes with poor convergence; however we rely instead on model assessment “fit” criteria of the parameter posteriors as described below to filter out poorly fit genes. The 90% credible interval (shortest interval containing 90% mass) is constructed from the empirical cumulative density of the posterior samples of each parameter using the R package *coda*.

For model assessment, i.e., to assess whether the data is captured well by our inferred models and satisfies our model assumptions including specific choices of distributions, we used concepts from the posterior predictive checking framework [23]. We specifically check the agreement between the distribution of observed data and of data generated from (“predicted by”) 100 inferred models, each of which was specified by parameters independently drawn from the posterior distribution, following the framework of Graphical posterior predictive checks but replacing the graphical check with a more quantitative AD-test check (Section 6.4 of [23]). The specific model assessment criterion we used required more than 75% of the models drawn to be capable of generating data samples statistically indistinguishable from the observed single- and 10-cell data (i.e., AD-test P ≥ 0.1). The Anderson-Darling or AD test is similar to the KS-test in that it tests if the same distribution could have yielded two sets of data samples (which in our case are the observed single- or k-cell samples and the same number of samples simulated from the inferred single- or k-cell model respectively). We use the ad.test implementation in the R package *kSamples*. In addition to filtering out genes that do not pass this AD-test criteria, we also excluded genes with extremely high variance of ON cells (posterior mean of *σ* greater than 5 in any of the two conditions) as part of model assessment. Genes whose inferred models pass these model assessment criteria in both conditions are referred to as *successfully modeled* genes, and genes that pass model assessment in a given condition are referred to as *successfully modeled* gene-condition models or gene-condition combinations (GCCs). Note that besides the default model assessment approach just described, our R package QVARKS also supports other model assessment options, including a single AD-test approach and posterior predictive pvalues (see Discussion above and vignette/manual provided with our R package).

When comparing two conditions for changes in an inferred quantity (either the parameter such as σ or a function of the parameters such as average overall expression of single-cells specified above as the log mean of *G*), we reduced the joint posterior distribution of the quantity in both conditions into a P-value. We chose to report P-values, instead of other hypotheses comparison measures such as the Bayes factor, to facilitate interpretation by researchers more familiar with classical “frequentist” statistical notions and to permit us to adjust P-values of all tested genes to account for multiple testing (using the Benjamini-Hochberg FDR procedure). We specifically converted the posterior probability that a parameter/variable in one condition is different than that in another condition to a P-value using a half-space approach [28]; this approach requires specification or estimation of the proportion of null hypotheses among all tested genes and we specify it conservatively at 100%. Several strengths of MCMC-based Bayesian inference are at play here in comparative assessment of heterogeneity in different conditions:

1. We are making inference about parameters of interest (*π*, *μ* and *σ*) in two conditions by averaging over uncertainty in the “nuisance” parameters related to detection behavior in a consistent fashion in both conditions (i.e., each MCMC sample is an eight-parameter configuration with detection parameters tied consistently to the same value across both conditions).
2. Posterior samples of the inferred parameters can be used to obtain posterior distribution of any function of the parameters, thereby allowing us to assess quantities such as the Shannon entropy based on the fraction of ON cells or the average overall expression of the entire cell population.
3. Bayesian approach provides a systematic way to incorporate *a priori* information available for any parameter (for instance, detection behavior parameters derived from a dilution-series experiment). For this study, we focused on non-informative priors to broaden the applicability of our strategy to settings where information from such *a priori* pilot experiments are not available.

### Macrophage single/10-cell data generation, normalization and downsampling

CD14+CD16- human peripheral blood monocytes were purified to >98% purity by magnetic bead negative selection (Dynabeads Monocyte selection kit, Invitrogen, Carlsbad, CA). These monocytes were aliquoted and frozen in 10% DMSO, 40% human serum, and 50% X-vivo 15. Frozen monocytes were thawed and differentiated into macrophages by *in vitro* culture on tissue culture-treated plastic dishes in X-Vivo-15 media (Lonza, Walkersville, MD) supplemented with 100 ng/mL macrophage-colony stimulating factor (M-CSF, R&D Systems, Minneapolis, MN) treatment over a period of 6 days with a full media change and M-CSF re-addition at day 3 and day 6. Fresh media on day 6 was supplemented with M-CSF + 100 ng/mL Interferon (IFN)γ and 100 ng/mL Tumor necrosis factor α (IFNγ+TNFα or IFNT; both from Peprotech, Rocky Hill, NJ), or M-CSF alone (Control or CNT). Cells were harvested for analysis 24 hours after treatment (day 7 of differentiation) by scraping in cold media.

We used fluorescence activated cell sorting (FACS) on a BD FACS-Aria II (Becton-Dickinson, Mountain View, CA) to sort single and ten cells into individual wells of 96-well low-profile PCR plates (Bio-rad, Richmond, CA) followed by reverse transcription and 18 cycles of specific-target pre-amplification using Celldirect one-step RT-PCR kit (Invitrogen). Preamplified cDNA was quantified using microfluidic qPCR (Taqman probe-based qPCR assays (Life Technologies, Carlsbad, CA) targeting 93 gene transcripts (selected based on their relevance in macrophage activation and core cellular processes (e.g., metabolism, RNA processing, core transcriptional and translational regulators) as well as representative genes from modules of co-expressed transcripts having myeloid-enriched expression patterns obtained using published gene expression data [29]) and three spike-in artificial control RNAs from the ERCC spike-in set (Life Technologies)) to comparatively assess cell-to-cell expression heterogeneity of human macrophages between CNT and IFNT conditions. The single-cell and 10-cell samples in each condition were profiled in four Fluidigm 96.96 plates (Fluidigm Corporation, South San Francisco, CA). To reduce technical confounding when comparing single cell responses between conditions, we FACS sorted cells from each of the conditions (CNT and IFNT conditions, as well as two other conditions from a related study) using a balanced distribution across multiple 96-well plates, followed by qPCR profiling.

QPCR was performed on a Fluidigm Biomark instrument using the normal speed cycling gene expression protocol. Data was exported from Fluidigm Real-time PCR Analysis software version 3.1.3, using Linear (Derivative) Baseline method, a global threshold of 0.01, and a 0.65 quality threshold, parameters which were found to exclude non-specific amplification and reduce plate-to-plate variation. We converted gene expression data exported from Fluidigm Real-time PCR Analysis software from Ct (Cycle threshold) units to the more convenient Et = 40-Ct units, as in previous studies [7], so that transcript copy counts can be approximated by 2^*Et*^ values upto a scaling factor as assumed in our model. Even if this copy count assumption does not hold exactly (or even approximately) for certain gene assays due to less than 100% qPCR efficiency, the model assessments performed after our MCMC-based Bayesian inference should automatically exclude such problematic assays. In the future, we could recover such gene assays if we know their qPCR efficiency (e.g., based on independent titration standards experiments) and use it to derive the copy counts instead of the 100% efficiency that is often assumed. We assigned samples where a gene is not detected an Et of–Infinity, and called them as non-detected samples (non-detects). After excluding samples with fewer than 10% of all assayed genes detected (as they may reflect wells that failed to amplify or receive a sorted cell), we had 84 single-cell and 88 10-cell samples available per condition for downstream analysis.

Since the single and 10-cell pool samples in CNT and IFNT conditions (along with samples in the two other conditions from a related study) had to be spread across 8 Fluidigm plates, all of which could not be run at the exact same time, we used spiked-in ERCC control RNA levels to track and correct for potential batch/plate effects. The ERCC control RNA were added at the same concentration to all the wells of each 96-well sorting plate prior to cell sorting, and we normalized all detected (i.e., Et > 0) gene measurements using the plate median of the ERCC spike-in with the highest Et value (ERCC-0003). In detail, we shifted all detected gene measurements in a plate by a plate-specific global factor, which is chosen such that the median ERCC-0003 level of the shifted data across all (non-standard) samples in the plate becomes the same across all plates. After removing plate effects using this plate-level normalization, the different plates’ data are concatenated to obtain one single/10-cell dataset per experiment.

In certain analyses where we had to compare the precision of SCKC against SC or KC (Fig 3), to ensure the same sample size (n = 80) across all three methods, we first randomly downsample the 84 single- and 88 ten-cell samples available per condition to 80 samples each per condition, and then run SCKC method on a random half of the single-cell and a random half of the 10-cell samples (repeating this random halving ten times to inspect run-to-run variation), and SC or KC method on all single- or all 10-cell samples respectively.

### RNA-seq data generation, processing and DE analysis

Note that to obtain the bulk population-level log_2_(FC) values, data from a related study was used. Similar experimental setup as described for generating our Fluidigm qPCR data was used to obtain and differentiate cells from donors, but instead of using qPCR on single- and 10-cell samples in CNT or IFNT (24 hour post stimulation) conditions, we analyzed bulk RNA material in CNT or IFNT (18 hours post stimulation) conditions from three different human donors using the Illumina TruSeq Ribozero RNA-Seq protocol with 500ng of total RNA according to manufacturer’s instructions. Standard RNA-Seq data analysis methods were used: Tophat2 [30] for splice-aware mapping, featureCounts [31] for counting reads mapped to gene exons, and DESeq2 [32] to perform the DE test and generate the log_2_(FC) values using the design”*gene expression ~ Donor + Treatment*” in R’s formula notation.

### Identifying differentially expressed (DE) genes using an approximate posterior-free method

We identified DE genes using the posterior probabilities converted to P-values as specified above. As described in the main text, we could also identify DE genes in a model-free or posterior-free fashion by directly testing for changes in k-cell expression mean in both conditions. The idea behind this test is that k-cell data is closer to bulk population-level data than single-cell data, and that bulk data when analyzed using t test or similar tests often is considered to yield more accurate (“ground-truth”) estimates of changes in overall expression of a gene (DE) than single-cell data (though this is not the case for estimating CHPs, where single- and k-cell data are valuable). But this posterior-free method treats all non-detects in the k-cell data as truly zero expression, which may not be valid for some lowly expressed genes or low values of k.

The posterior-free method uses a linear regression model of each gene’s k-cell measurement across two conditions against these dependent variables: *treatment* to model fold change between the two conditions, *ERCC expression* to adjust for potential well effects, and *plate variable* to adjust for potential plate effects. Non-detects are assumed to be have zero expression. In R notation, the linear model is “*k-cell expression of a given gene ~ treatment + ERCC-0002-expression + ERCC-0003-expression + ERCC-0044-expression + plate*”. We extract the P-values of the significance of the *treatment* coefficients (log fold change between two conditions such as IFNT vs. CNT) being different from zero, and adjust them for multiple testing using the Benjamini-Hochberg procedure. Only genes at adjusted P or adjP < 0.05 are declared as DE hits.

### Simulation scenarios and posterior surface scanning

We simulated datasets under different levels of biological and technical variation, thereby translating to different levels of difficulty when inferring the true parameters. We first explain this procedure for single-condition simulations and then outline changes to the procedure for comparative two-condition simulations. The mean of ON-cells *μ* is set at 10 Et units in all scenarios, whereas the other parameters are set at

1. *π* = 0.8, *σ* = 0.5 for the low inference difficulty,
2. *π* = 0.5, *σ* = 1.0 for the medium inference difficulty and
3. *π* = 0.2, *σ* = 2.0 for the high inference difficulty scenarios.

These three biological variation scenarios are illustrated in S1A Fig.

The detection efficiency of the gene assay as given by the logistic function is also set at three levels. Specifically we set the logistic function’s parameters at:

1. *c* = *μ* − *σ*, *m* = *σ*/4 for a good assay that detects 82% of single-cell samples on average,
2. *c* = *μ*, *m* = *σ*/4 for a medium assay that detects 50% of single-cell samples on average, and
3. *c* = *μ* + *σ*, *m* = *σ*/4 for a bad assay that detects 18% of single-cell samples on average.

These settings of the logistic “assay” leads to negligible loss of k-cell samples on average, since the mean k-cell expression is k-fold higher than mean single-cell expression and the slope *m* of the logistic function is set above such that the detection probability changes from 0 to 100% in a steep fashion as a function of the increasing expression strength. Note that this likely favors the k-cell only approach when comparing its performance against our single/k-cell strategy (since assay drop-offs may occur for certain genes in real k-cell data when k is smaller). Larger values of *m* that lead to a more gradual increase in detection probability with expression increase (and hence drop-off or loss in both single/k-cell samples) could also be tested for simulations in future, if dilution standards experiments of typical gene assays have this detection behavior.

Besides noise from imperfect detection of gene transcripts, there could be additional experimental noise when measuring gene expression [12]. So we ran our simulations under four realistic configurations of measurement noise and a reference configuration of no additional noise. The four realistic configurations were based on quantitative measures of total technical noise in our experimental setup (encompassing all noise sources such as efficiency, amplification and sampling noise [12]) derived from a dilution series experiment conducted alongside our macrophage single/k-cell experiments. This titration data exhibited similar patterns of dependency between the level of total noise and mean expression (S1B Fig) as previously observed [12], and thereby helped select these noise settings:

1. [C0.5] Constant white noise sampled from a Gaussian distribution with a mean 0 and standard deviation (std) of 0.5 Et is added to the simulated Et value of detected single/k-cell expression samples (the “detects” simulated under one of the “3 biological variation x 3 assay sensitivity” scenarios above). This scenario is representative of noise characteristics of highly expressed genes in our experiment, whose measurements exhibit similar noise levels in both single- and k-cell measurements (the plateau region in S1B Fig, with 0.5 Et chosen for std to represent gene assays that are more challenging than the median gene in the figure with 0.25 Et noise levels).
2. [C1] Constant white noise of std = 1 Et is added to test how the different methods behave under high levels of noise.
3. [T0.5,0.25] A two-step noise that adds a white noise of std = 0.5 Et to single-cell detects and std = 0.25 Et to k-cell detects. This corresponds to the transition in S1B Fig and captures the notion that single-cell samples are typically measured with higher technical noise than k-cell samples due to lower transcript levels in the former. Please note that since the mean-noise relation in S1B Fig can differ substantially from gene to gene (and was extrapolated to expression levels not observed in the experiment), we opted to employ a simpler two-step noise configuration instead of the observed mean-noise relation to account for these uncertainties.
4. [T1,0.5] Another two-step noise that adds a white noise of std = 1 Et to single-cell detects and std = 0.5 Et to k-cell detects.
5. [No white noise] Reference configuration that adds no additional noise to single/k-cell detects.

We simulate single-cell/k-cell data (SC/KC) under any of the above “3 biological variation x 3 assay sensitivity x 5 noise configuration” scenarios = 45 settings using our model, and infer back the true values using only the simulated data. We could have used our MCMC procedure for inference, but wanted to rule out potential differences in the MCMC convergence behavior of the SC, KC or SCKC approaches from confounding the intrinsic differences among these methods. So we opted for a simpler posterior surface scanning procedure that involved computing the posterior in each combination of the parameter values listed below and using this coarse grid of likelihood values to approximate the posterior distribution and calculate its mode and 90% CrI (credible interval) reported in Fig 2 and S2 and S3 Figs discussed in the main text. The posterior surface is scanned at the grid points defined by these parameter values:

1. Vary π from 0 to 1 in steps of 0.02.
2. Vary μ from 0 to 40 in in steps of 1 Et unit.
3. Vary σ from 0 to 5 in steps of 0.1 Et unit.
4. Set c,m to the true value, since we assume it is known to the inference procedure. This is a realistic assumption since data from titration experiments of known mRNA inputs can be used to independently determine these parameters.

Note that the posterior (derived from single-cell or Fenton-Wilkinson approximated k-cell likelihoods) is a function of just the five parameters above and not of any additional measurement noise configuration parameter, since our model is unaware of the additional measurement noise added to simulated data.

We performed the comparative two-condition simulations similar to the single-condition simulations described above (e.g., under the same 3 assay sensitivity and 5 noise configuration settings), but with the following changes. Since we need to assess the performance of different methods in identifying changes in heterogeneity parameters between two conditions (as opposed to inferring the parameter values within one condition as in the three single-condition biological variation scenarios), we now tested three comparative scenarios. A gene has

1. 80% vs. 50% ON cells (*π* of 0.8 vs. 0.5) between the two conditions,
2. *π* of 0.6 vs. 0.4, and
3. *π* of 0.5 vs. 0.2.

In all these scenarios, the ON cells have a mean (*μ*) of 10 Et and a standard deviation (*σ*) of 1 Et in both conditions, so that both conditions are simulated using an assay with the same logistic detection behavior (note that the logistic parameters in our simulations are derived from *μ*, *σ* as described above). Grid-based posterior scan is now run separately on both conditions of a simulated gene to approximate the posterior distribution of a parameter in each condition, and the two resulting distributions jointly analyzed to approximate the posterior of the difference in the parameter between the conditions. Relevant code snippets implementing the single/two-condition simulations are provided (see Data Availability for download URL).

## Supporting Information

S1 Fig Inference difficulty and measurement noise of simulation scenarios. Three levels of inference difficulty were used for single-condition simulations and four configurations of measurement noise (and one more configuration of no noise) were used for both single- and two-condition simulations. These configurations, in combination with different assay sensitivity (detection) settings, yield the diverse simulation scenarios employed to compare the performance of SC, KC and SCKC methods. A. Levels of inference difficulty when simulating gene expression profiles. Low inference difficulty simulation scenario for instance corresponds to a gene with a high fraction of ON cells with tight expression distribution. B. Measurement noise configurations when simulating gene expression profiles. Technical noise vs. average expression relationship observed in a dilution-series experiment was used to define the four realistic noise configurations for the simulation analyses. The estimate of total technical noise (including amplification, efficiency and sampling noise) when measuring different genes (different colored lines) at different dilutions of a standard bulk mRNA sample is shown. The average expression of the gene (x axis) is plotted against the standard deviation of the technical replicate measurements (y axis). The dilution series experiment was done using bulk mRNA pooled from human macrophages residing in diverse conditions in a related study—a total of seven dilutions were performed spanning a range of medium to high mRNA concentrations, and each dilution had eight technical replicates (except for one dilution which had only seven replicates due to an outlying measurement). Only genes that pass our quality control criteria are shown here: 1) The gene must exhibit a range of detection behaviors along the standard curve, or specifically its non-detect frequency should be at least 0.7 at the lowest concentration and at most 0.1 at the highest concentration (with the concentrations with zero or unity non-detect frequencies ignored for further analysis), and 2) the measured non-detect frequency and Et value of the gene at different concentrations should be concordant, or specifically the baseline Et value corresponding to single transcript copy detection (estimated using Poisson statistics from both the non-detect frequency and measured Et value at each concentration as in digital PCR or digital RNA-Seq (Grün et al. 2014, Nat. Methods 11, 637–640 [12]) should not vary by more than 0.5 standard deviation units across different concentrations. Note that this baseline Et value estimate, averaged across different concentrations, is subtracted from the average measured Et value of a gene at every concentration to obtain the average gene expression shown in x axis. Also shown is a local regression fit along with its 95% confidence level band (visualized using R package *ggplot2*’s stat_smooth function with default options).
(TIF)

S2 Fig SC *vs*. KC *vs*. SCKC performance for single-condition simulations. Single-condition simulation results for sample size n = 1000 shown in main text Fig 2 is repeated here, but additional results on KC vs. SC comparison and additional simulation scenarios pertaining to medium inference difficulty and medium assay sensitivity are shown. All other aspects of this figure including x/y-axis format are same as main text Fig 2 - for instance, parameter inferences (posterior mode and 90% CrI (Credible Interval) based on a grid-based posterior scan) were made for ten simulated datasets (sample size n = 1000) and averaged before display for each simulation scenario and method. Guide to understand the plots is also shown alongside the legends. A. Simulation results for single-condition parameter inferences, where each dot corresponds to an inferred parameter for a gene simulated under one condition according to a simulation scenario. Note that SC provides worse estimates than SCKC or KC under all noise configurations for most of the high-difficulty scenarios (red symbols), and better estimates with lower error particularly for σ in some noise settings for the medium/low-difficulty scenarios (blue/green symbols); but this advantage of SC disappears in a two-condition, comparative simulation setup (S3 Fig) where error in the two conditions tend to cancel out (S4 Fig). B. Simulation results shown in (A) are copied here, but the shapes of dots (simulation scenario genes) now indicate measurement noise configuration instead of assay sensitivity of the simulation scenarios.
(TIF)

S3 Fig SC *vs*. KC *vs*. SCKC performance for two-condition simulations. Simulation results for sample size n = 1000 on assessing CHP differences between two conditions under different assay sensitivity and noise configurations. This figure is similar to the above S2 Fig in its x/y-axis format and legend, except that the estimate of the parameter difference between two conditions is shown here instead of the single-condition parameter estimate. Guide to understand the plots is also shown alongside the legends. A. Simulation results for the comparative two-condition parameter inferences, where each dot corresponds to condition-specific change in inferred parameter for a gene simulated under two conditions according to a simulation/comparison scenario. Note that SCKC performs comparable to KC in most settings, with better performance for large k (lighter symbols), similar to what was observed in the single-condition simulations but to a lesser extent (in terms of absolute error advantages, since errors in both conditions tend to cancel out in a comparative analysis as shown in S4 Fig). B. Simulation results shown in (A) are copied here, but the shapes of dots (simulation/comparison scenario genes) now indicate measurement noise configuration instead of assay sensitivity of the simulation scenarios. This shows for instance that KC is better than SCKC in the T0.5,0.25 noise setting where a very low amount of measurement noise is added to the k-cell data.
(TIF)

S4 Fig Bias/error in parameter estimates in two- *vs*. one- condition simulation settings. Error in parameter estimates is significantly mitigated in two-condition compared to one-condition simulation settings, particularly for the σ parameter. The x-axis is showing the absolute error of a parameter (difference between its inferred and true value), averaged across both conditions and all ten simulation runs; the y-axis is showing the absolute error of the parameter difference between the two conditions, averaged across all ten simulation runs. Both x- and y- axis are divided (scaled) by the true value of the parameter difference between the two conditions (if non-zero).
(TIF)

S5 Fig SC *vs*. KC *vs*. SCKC precision comparisons at similar bias/error on real data. Experimental data were generated from untreated (CNT) and IFN+TNF-treated (IFNT) human macrophages, and the three methods assessed on this data. Density plots of parameter inferences for all gene-condition combinations (GCCs) successfully modeled by all three assessed methods are shown. Each plot compares the performance of the two indicated methods as follows: The x-axis indicates the difference in the two methods’ estimates (posterior means); the y-axis is the difference in the CrI width of the two methods’ estimates; and both x- and y- axis values are divided (scaled) by the average parameter estimate if non-zero (specifically posterior mean estimate, averaged across SC, KC and SCKC methods, and used in place of the unknown true parameter value). To compare two methods when ground truth is not known (see also text), we only consider GCCs whose parameter estimates are similar between the compared methods (shown as red dots within the x = -0.2 to 0.2 red band i.e., GCCs whose estimates by the two methods differ by less than 20% of the average parameter estimate). Shown alongside are the histogram of red dots and the (method1 vs method2) percentages indicating the percentage of GCCs inside the red band whose method1 (or method2) CrI is tighter than that of the other method by at least the same band width of 0.2 units (these histograms and percentages are also shown in main text Fig 3A, as the same downsampling for SCKC chosen in Fig 3A is chosen here as well).
(TIF)

S6 Fig Comparison of QVARKS, MAST and SCDE methods on differential expression (DE). Different methods were applied on our macrophage IFNT vs. CNT data and evaluated by checking if their estimated log_2_-fold-change (log_2_(FC)) in average gene expression between IFNT vs. CNT conditions are correlated (A-B) or close (C-D) to the “ground truth” log_2_(FC) estimated from bulk RNA-Seq data. These correlations are shown for different values of bulk RNA-Seq log_2_(FC) cutoffs (x axis) (i.e., for each cutoff value, the correlation is only computed across the starting set of genes that satisfy “abs(bulk log_2_(FC)) ≤ cutoff”). We similarly compute the closeness to true values, quantified conversely by the average squared error (square of the difference between the bulk RNA-Seq log_2_(FC) value and the log_2_(FC) value estimated by a given method, averaged across the genes under consideration). We first evaluate all methods using a starting set of 55 genes that have more than 5 observed ON cells in both conditions (the “ON-cells filter” that ensures sufficient data is available to estimate QVARKS parameters). We also evaluate our QVARKS methods on other starting gene sets obtained by combining the “ON-cells filter” with two other filters: i) genes whose log_2_(FC) CrI width estimated by our method is at most 5 units, so that the point estimate of DE used in these comparisons are reliable, and ii) genes that passed our AD-test based model assessment criteria. A. QVARKS SCKC and KC correlation to bulk RNA-Seq DE is comparable to that of MAST and SCDE across all scenarios, and QVARKS SC performs comparable to other methods once CrI filter is used to remove genes with large uncertainty in their estimates. B. QVARKS SCKC correlation to bulk RNA-Seq DE using ten random 50% downsamplings of the dataset (the same downsamplings as in main text Fig 3 to make QVARKS SCKC the same sample size as QVARKS SC or KC) shows the extent of run-to-run or sampling variation in our DE performance metric. C. Same as (A) but showing average squared error instead of Pearson correlation. All input modes of QVARKS perform better than MAST and SCDE. D. Same as (B) but showing average squared error instead of Pearson correlation.
(TIF)

S7 Fig Checking the concordance of single-/10-cell average expression. Average expression derived from single-cell data tended to be consistently lower than that obtained from 10-cell data (divided by 10) for transcripts expressed at medium or low levels. Note that average expression here refers to average of the 2^Et^ measurement values, after assuming non-detects as zero expression (i.e., setting their 2^Et^ to 0).
(TIF)

S8 Fig Posterior-based vs. posterior-free differential expression. For the genes successfully modeled in both IFNT and CNT conditions by our approach, we show the log_2_ fold change of their bulk/overall mean expression between the two conditions and the list of significant differentially expressed (DE) genes at adjusted P < 0.05, as determined by two methods. Though the DE results from both methods are not expected to be exactly equal (as explained in Methods), the concordance between them as shown here reinforces each method’s discoveries.
(TIF)

S9 Fig Changes in heterogeneity parameters are largely robust against well-to-well technical noise. We augmented our method with an additional pre-processing/normalization step that accounts for one of the key noise sources: well-to-well variation in sampling efficiency (inspired by the “Model 1” strategy used in (Grün et al. 2014, Nat. Methods 11, 637–640 [12])). This normalization involved inferring this noise using the Et values of three ERCC control mRNAs spiked in at the same concentration across wells, and then removing the effect of this noise from the data and applying our Bayesian inference procedure on the corrected data. A. Technical factors contributed to well-to-well variations. The mean Et value of the three control ERCC spike-in mRNAs is significantly correlated with the median of highly expressed genes across wells, both within and across plates, suggesting that well-to-well differences in the starting amount of mRNA, or sampling efficiency, or other related technical factors likely contributed substantially to well-to-well variations. This plot also shows that the magnitude of this particular noise is not large in our data. Here a highly expressed gene is defined as a gene with non-zero expression level in at least 90% of all single/k-cell samples measured in either condition. Also shown are robust linear regression fits along with 95% confidence level bands (visualized using R package *ggplot2*’s stat_smooth function with “rlm” method and default options). B. The changes in parameter estimates between IFNT vs. CNT are largely similar before or after performing the new per-well normalization designed to remove well-to-well variations shown in (A) (the black line is the x = y diagonal line). This comparison is done using the 39 overlapping genes out of the 41 and 47 genes passing model assessment (in both CNT and IFNT conditions) in our macrophage data before and after performing the per-well normalization respectively. Note that the per-well normalization factor subtracted from each gene measurement within a well is a weighted sum of the three ERCC levels in the well, where the weights are the corresponding coefficients of a linear regression model fitted separately for single/k-cell data in each condition (in R notation: “Median expression of highly expressed genes ∼ ERCC-0002 + ERCC-0003 + ERCC-0044”).
(TIF)

S10 Fig Alternate notions of discrete heterogeneity. The Shannon entropy function computed using π (entropy(π) = −π log_2_(π) − (1 − π)log_2_(1 − π)) captures the intuitive notion that a cell population containing a balanced number of ON and OFF cells is more heterogeneous than another where almost all cells are ON or almost all are OFF. A. Inferred alterations in entropy in IFNT treatment relative to CNT is shown for the genes successfully modeled in both conditions. The 23 differentially expressed genes identified using overall expression changes, along with whether they are increased or decreased, is also indicated. The 90% Crl is indicated by the lightly shaded lines around the mean of the posterior distribution. B. Genes with significant (adjP < 0.05) changes in entropy between the conditions (and gene RELA) are shown along the entropy function’s curve.
(TIF)

S11 Fig Mean-variation relationship and related checks. Linear regression fit of changes in continuous variation (σ) as a function of changes in the average expression among ON cells (µ; A) or ON rate (π; B) between IFNT and CNT conditions using the genes successfully modeled in both conditions. For genes with significant alterations in σ (from main text Fig 6A and labeled here), changes in σ cannot simply be explained by differences in μ or π between the conditions. Confidence bands around the linear regression fits are at 95% confidence level (and visualized using R package *ggplot2*’s stat_smooth function with “lm” method and default options).
(TIF)

S12 Fig Robustness of Differential Heterogeneity (DH). For genes with significant alterations in σ (DH genes from main text Fig 6A, with adjusted Pvalues reported here), difference in σ was non-zero for many of the ten random 50% downsamplings of the dataset (the same downsamplings as in main text Fig 3). Also shown here for comparison are the inferences made by QVARKS on two non-overlapping subsets of the data: single-cell (SC) samples alone vs. k-cell (KC) samples alone. The 90% Crl is indicated by the line around the mean of the posterior distribution.
(TIF)
