# Supplementary figures and images for: Robust Inference of Cell-to-Cell Expression Variations from Single- and K-Cell Profiling
Source: PLoS Comput Biol. 2016 Jul 20;12(7):e1005016. doi: 10.1371/journal.pcbi.1005016 (PMC4954693; doi:10.1371/journal.pcbi.1005016)

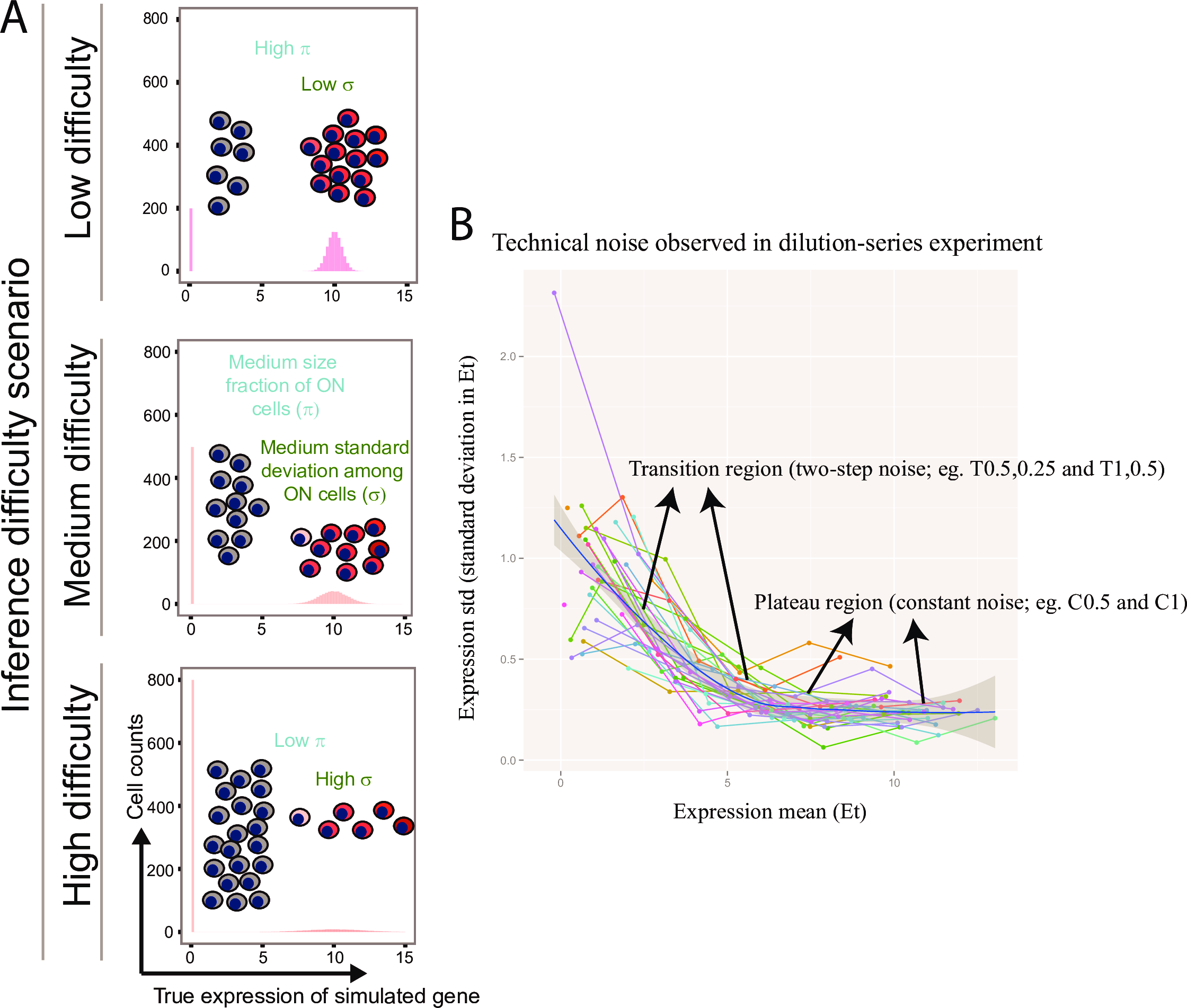

Supplement: S1 Fig — Three levels of inference difficulty were used for single-condition simulations and four configurations of measurement noise (and one more configuration of no noise) were used for both single- and two-condition simulations. These configurations, in combination with different assay sensitivity (detection) settings, yield the diverse simulation scenarios employed to compare the performance of SC, KC and SCKC methods. A. Levels of inference difficulty when simulating gene expression profiles. Low inference difficulty simulation scenario for instance corresponds to a gene with a high fraction of ON cells with tight expression distribution. B. Measurement noise configurations when simulating gene expression profiles. Technical noise vs. average expression relationship observed in a dilution-series experiment was used to define the four realistic noise configurations for the simulation analyses. The estimate of total technical noise (including amplification, efficiency and sampling noise) when measuring different genes (different colored lines) at different dilutions of a standard bulk mRNA sample is shown. The average expression of the gene (x axis) is plotted against the standard deviation of the technical replicate measurements (y axis). The dilution series experiment was done using bulk mRNA pooled from human macrophages residing in diverse conditions in a related study—a total of seven dilutions were performed spanning a range of medium to high mRNA concentrations, and each dilution had eight technical replicates (except for one dilution which had only seven replicates due to an outlying measurement). Only genes that pass our quality control criteria are shown here: 1) The gene must exhibit a range of detection behaviors along the standard curve, or specifically its non-detect frequency should be at least 0.7 at the lowest concentration and at most 0.1 at the highest concentration (with the concentrations with zero or unity non-detect frequencies ignored for further [file pcbi.1005016.s001.tif]

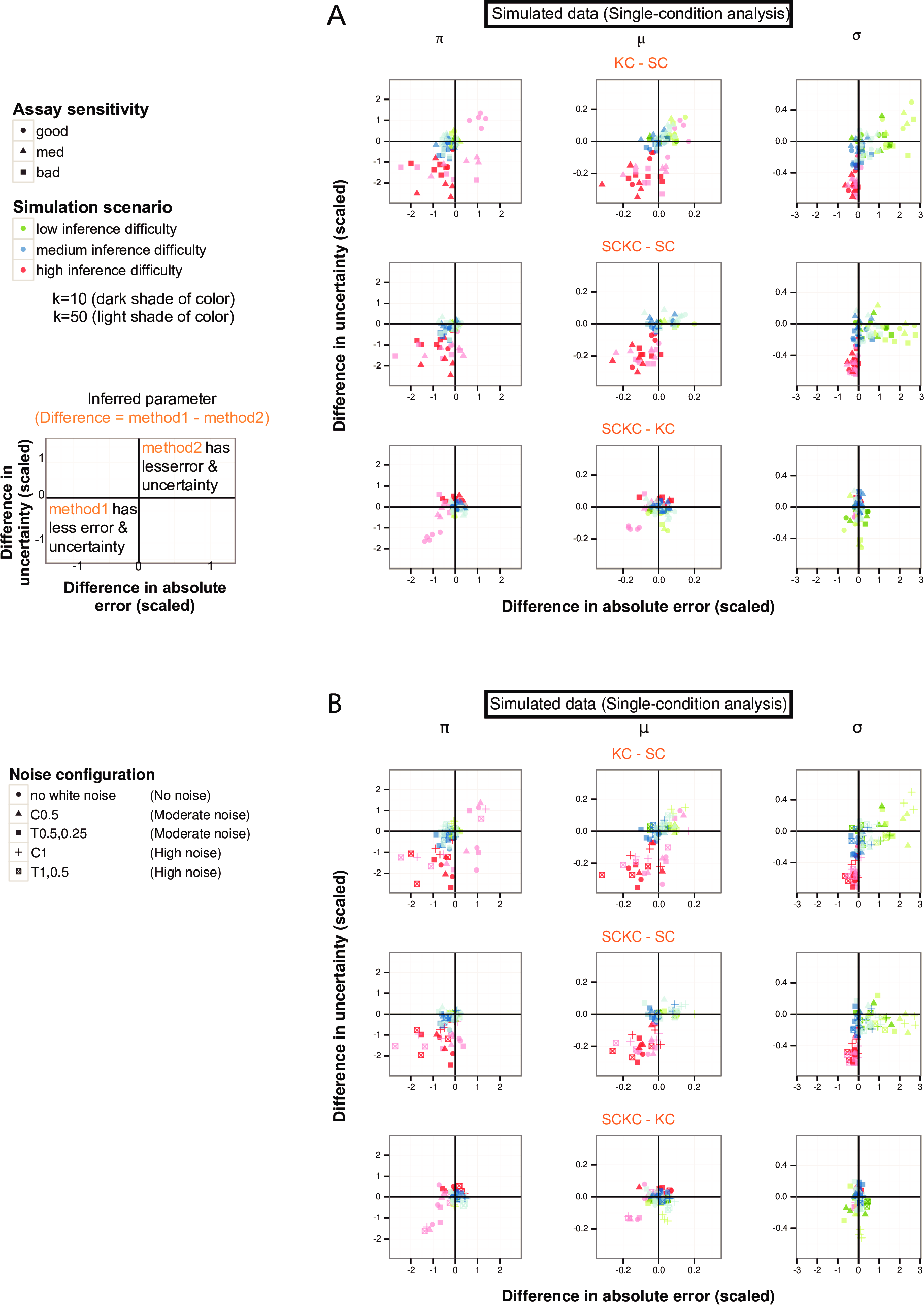

Supplement: S2 Fig — Single-condition simulation results for sample size n = 1000 shown in main text Fig 2 is repeated here, but additional results on KC vs. SC comparison and additional simulation scenarios pertaining to medium inference difficulty and medium assay sensitivity are shown. All other aspects of this figure including x/y-axis format are same as main text Fig 2 - for instance, parameter inferences (posterior mode and 90% CrI (Credible Interval) based on a grid-based posterior scan) were made for ten simulated datasets (sample size n = 1000) and averaged before display for each simulation scenario and method. Guide to understand the plots is also shown alongside the legends. A. Simulation results for single-condition parameter inferences, where each dot corresponds to an inferred parameter for a gene simulated under one condition according to a simulation scenario. Note that SC provides worse estimates than SCKC or KC under all noise configurations for most of the high-difficulty scenarios (red symbols), and better estimates with lower error particularly for σ in some noise settings for the medium/low-difficulty scenarios (blue/green symbols); but this advantage of SC disappears in a two-condition, comparative simulation setup (S3 Fig) where error in the two conditions tend to cancel out (S4 Fig). B. Simulation results shown in (A) are copied here, but the shapes of dots (simulation scenario genes) now indicate measurement noise configuration instead of assay sensitivity of the simulation scenarios. (TIF) [file pcbi.1005016.s002.tif]

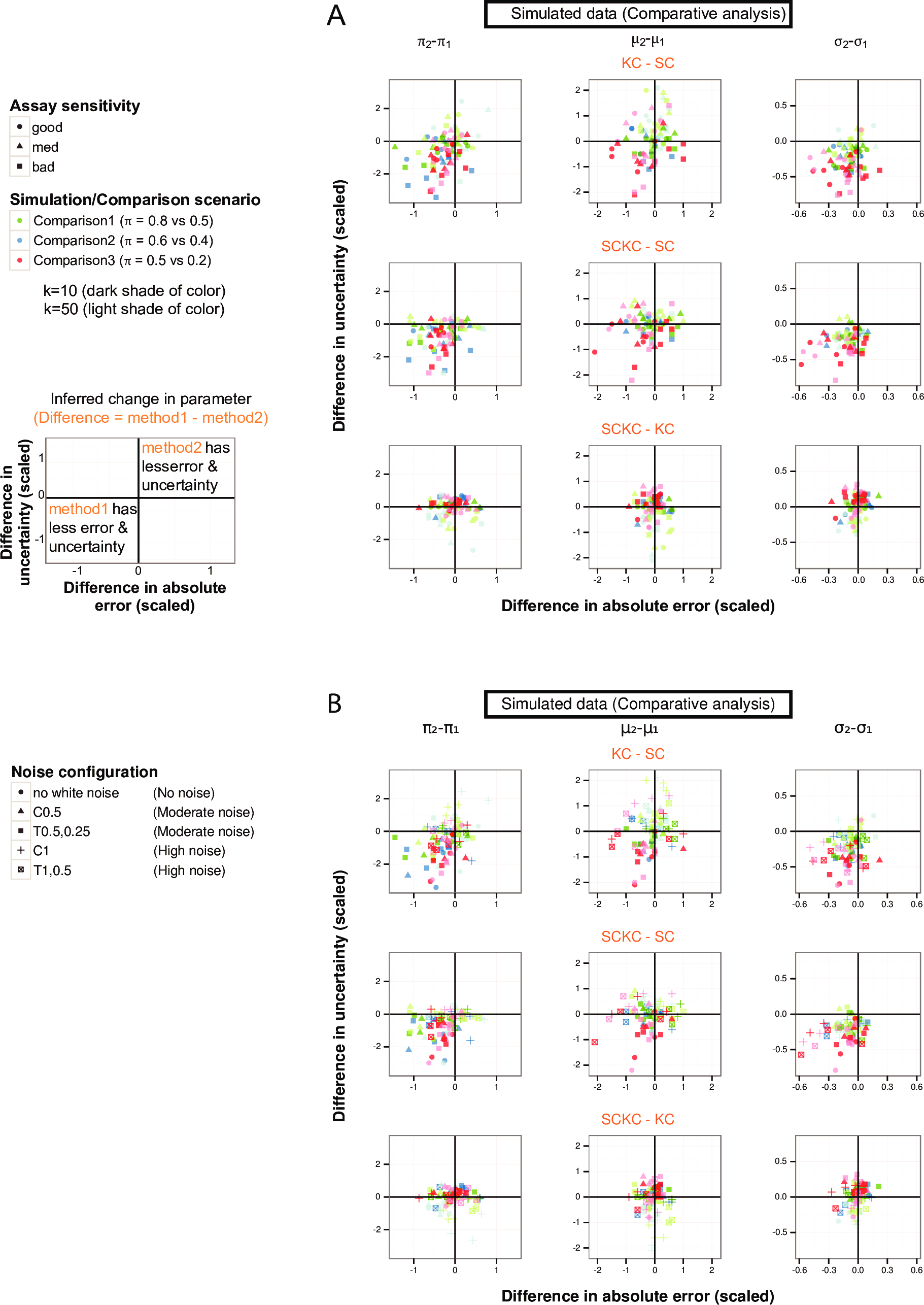

Supplement: S3 Fig — Simulation results for sample size n = 1000 on assessing CHP differences between two conditions under different assay sensitivity and noise configurations. This figure is similar to the above S2 Fig in its x/y-axis format and legend, except that the estimate of the parameter difference between two conditions is shown here instead of the single-condition parameter estimate. Guide to understand the plots is also shown alongside the legends. A. Simulation results for the comparative two-condition parameter inferences, where each dot corresponds to condition-specific change in inferred parameter for a gene simulated under two conditions according to a simulation/comparison scenario. Note that SCKC performs comparable to KC in most settings, with better performance for large k (lighter symbols), similar to what was observed in the single-condition simulations but to a lesser extent (in terms of absolute error advantages, since errors in both conditions tend to cancel out in a comparative analysis as shown in S4 Fig). B. Simulation results shown in (A) are copied here, but the shapes of dots (simulation/comparison scenario genes) now indicate measurement noise configuration instead of assay sensitivity of the simulation scenarios. This shows for instance that KC is better than SCKC in the T0.5,0.25 noise setting where a very low amount of measurement noise is added to the k-cell data. (TIF) [file pcbi.1005016.s003.tif]

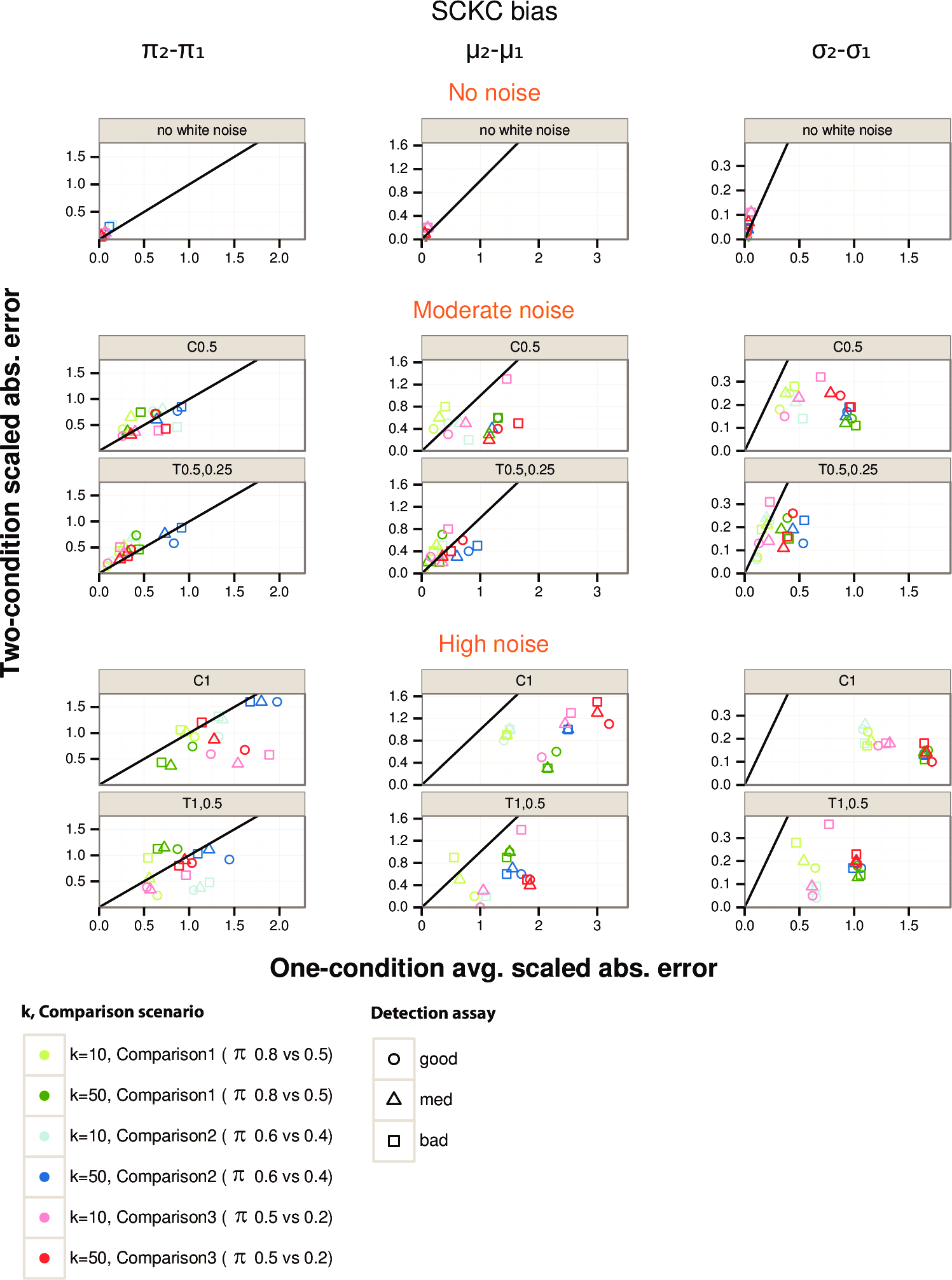

Supplement: S4 Fig — Error in parameter estimates is significantly mitigated in two-condition compared to one-condition simulation settings, particularly for the σ parameter. The x-axis is showing the absolute error of a parameter (difference between its inferred and true value), averaged across both conditions and all ten simulation runs; the y-axis is showing the absolute error of the parameter difference between the two conditions, averaged across all ten simulation runs. Both x- and y- axis are divided (scaled) by the true value of the parameter difference between the two conditions (if non-zero). (TIF) [file pcbi.1005016.s004.tif]

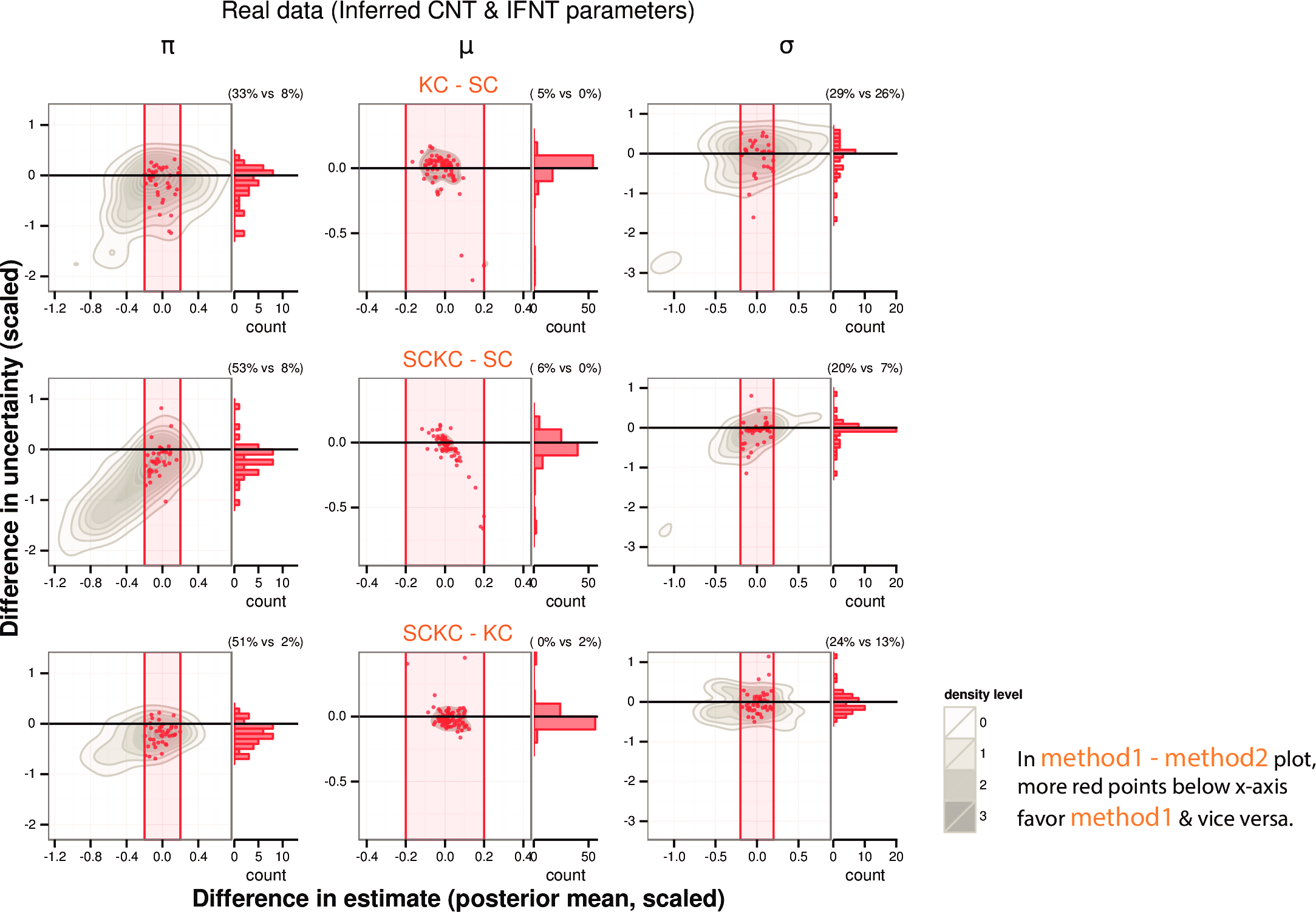

Supplement: S5 Fig — Experimental data were generated from untreated (CNT) and IFN+TNF-treated (IFNT) human macrophages, and the three methods assessed on this data. Density plots of parameter inferences for all gene-condition combinations (GCCs) successfully modeled by all three assessed methods are shown. Each plot compares the performance of the two indicated methods as follows: The x-axis indicates the difference in the two methods’ estimates (posterior means); the y-axis is the difference in the CrI width of the two methods’ estimates; and both x- and y- axis values are divided (scaled) by the average parameter estimate if non-zero (specifically posterior mean estimate, averaged across SC, KC and SCKC methods, and used in place of the unknown true parameter value). To compare two methods when ground truth is not known (see also text), we only consider GCCs whose parameter estimates are similar between the compared methods (shown as red dots within the x = -0.2 to 0.2 red band i.e., GCCs whose estimates by the two methods differ by less than 20% of the average parameter estimate). Shown alongside are the histogram of red dots and the (method1 vs method2) percentages indicating the percentage of GCCs inside the red band whose method1 (or method2) CrI is tighter than that of the other method by at least the same band width of 0.2 units (these histograms and percentages are also shown in main text Fig 3A, as the same downsampling for SCKC chosen in Fig 3A is chosen here as well). (TIF) [file pcbi.1005016.s005.tif]

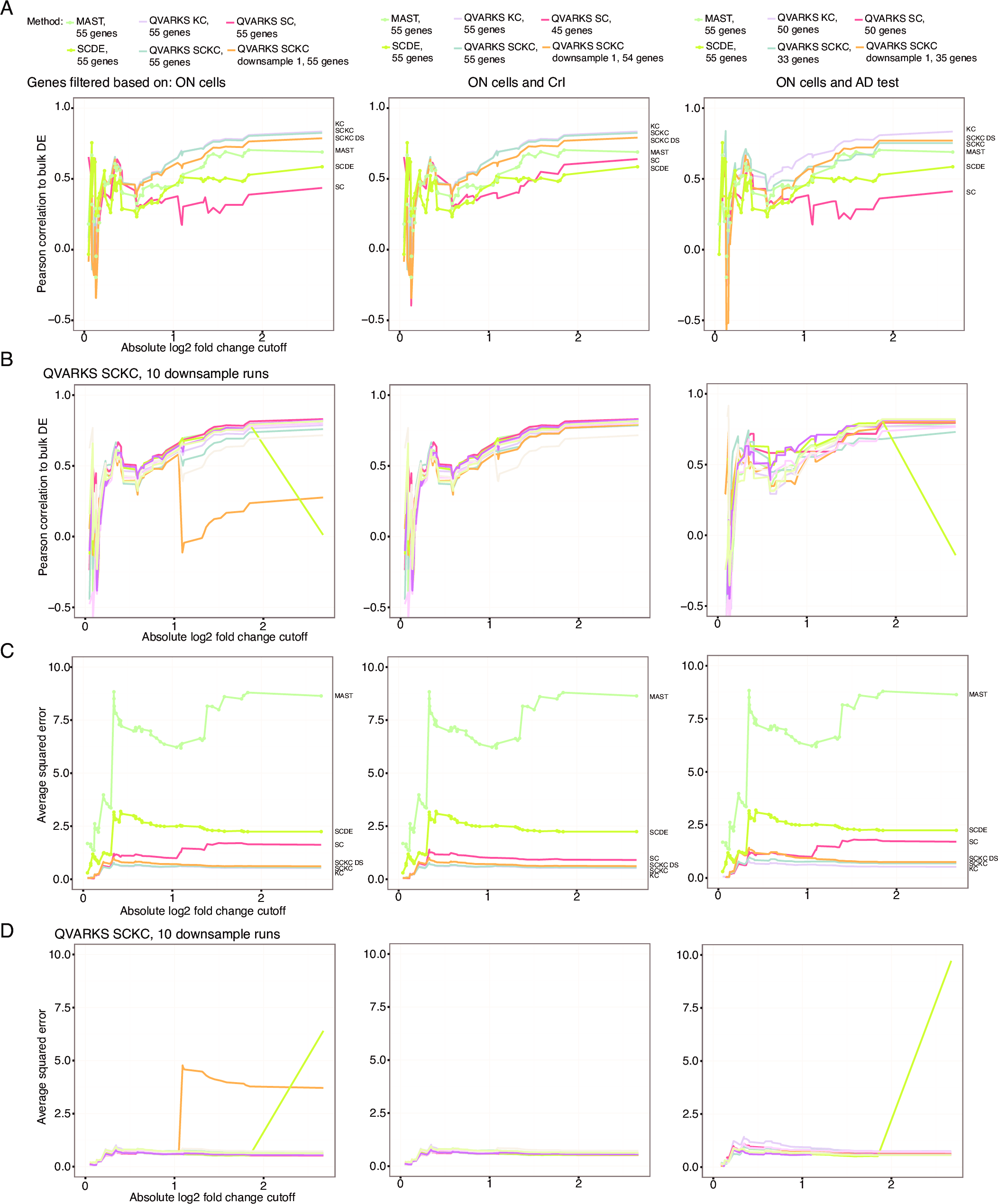

Supplement: S6 Fig — Different methods were applied on our macrophage IFNT vs. CNT data and evaluated by checking if their estimated log2-fold-change (log2(FC)) in average gene expression between IFNT vs. CNT conditions are correlated (A-B) or close (C-D) to the “ground truth” log2(FC) estimated from bulk RNA-Seq data. These correlations are shown for different values of bulk RNA-Seq log2(FC) cutoffs (x axis) (i.e., for each cutoff value, the correlation is only computed across the starting set of genes that satisfy “abs(bulk log2(FC)) ≤ cutoff”). We similarly compute the closeness to true values, quantified conversely by the average squared error (square of the difference between the bulk RNA-Seq log2(FC) value and the log2(FC) value estimated by a given method, averaged across the genes under consideration). We first evaluate all methods using a starting set of 55 genes that have more than 5 observed ON cells in both conditions (the “ON-cells filter” that ensures sufficient data is available to estimate QVARKS parameters). We also evaluate our QVARKS methods on other starting gene sets obtained by combining the “ON-cells filter” with two other filters: i) genes whose log2(FC) CrI width estimated by our method is at most 5 units, so that the point estimate of DE used in these comparisons are reliable, and ii) genes that passed our AD-test based model assessment criteria. A. QVARKS SCKC and KC correlation to bulk RNA-Seq DE is comparable to that of MAST and SCDE across all scenarios, and QVARKS SC performs comparable to other methods once CrI filter is used to remove genes with large uncertainty in their estimates. B. QVARKS SCKC correlation to bulk RNA-Seq DE using ten random 50% downsamplings of the dataset (the same downsamplings as in main text Fig 3 to make QVARKS SCKC the same sample size as QVARKS SC or KC) shows the extent of run-to-run or sampling variation in our DE performance metric. C. Same as (A) but showing average squared error instead of Pearson correlation. All input m [file pcbi.1005016.s006.tif]

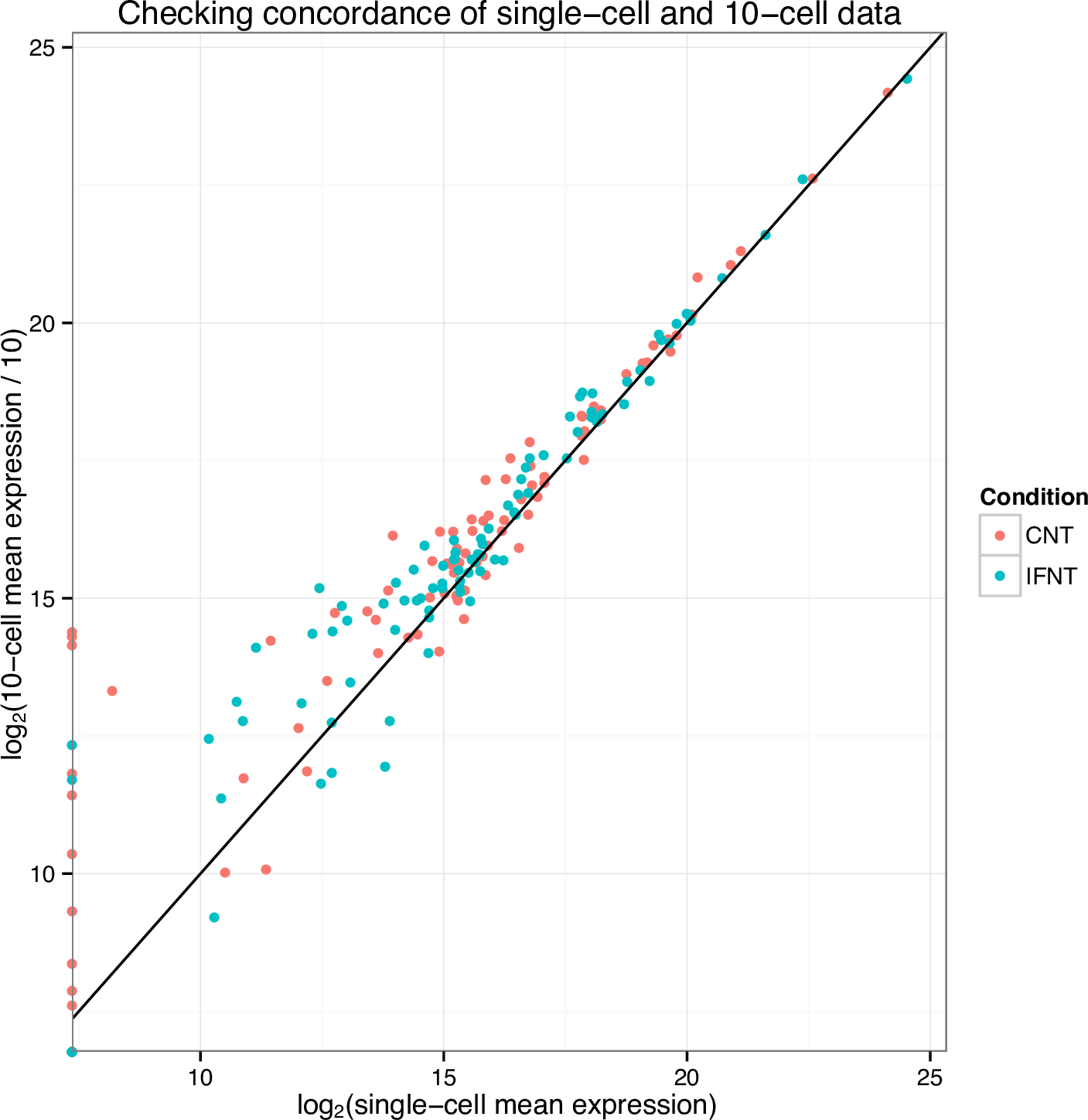

Supplement: S7 Fig — Average expression derived from single-cell data tended to be consistently lower than that obtained from 10-cell data (divided by 10) for transcripts expressed at medium or low levels. Note that average expression here refers to average of the 2Et measurement values, after assuming non-detects as zero expression (i.e., setting their 2Et to 0). (TIF) [file pcbi.1005016.s007.tif]

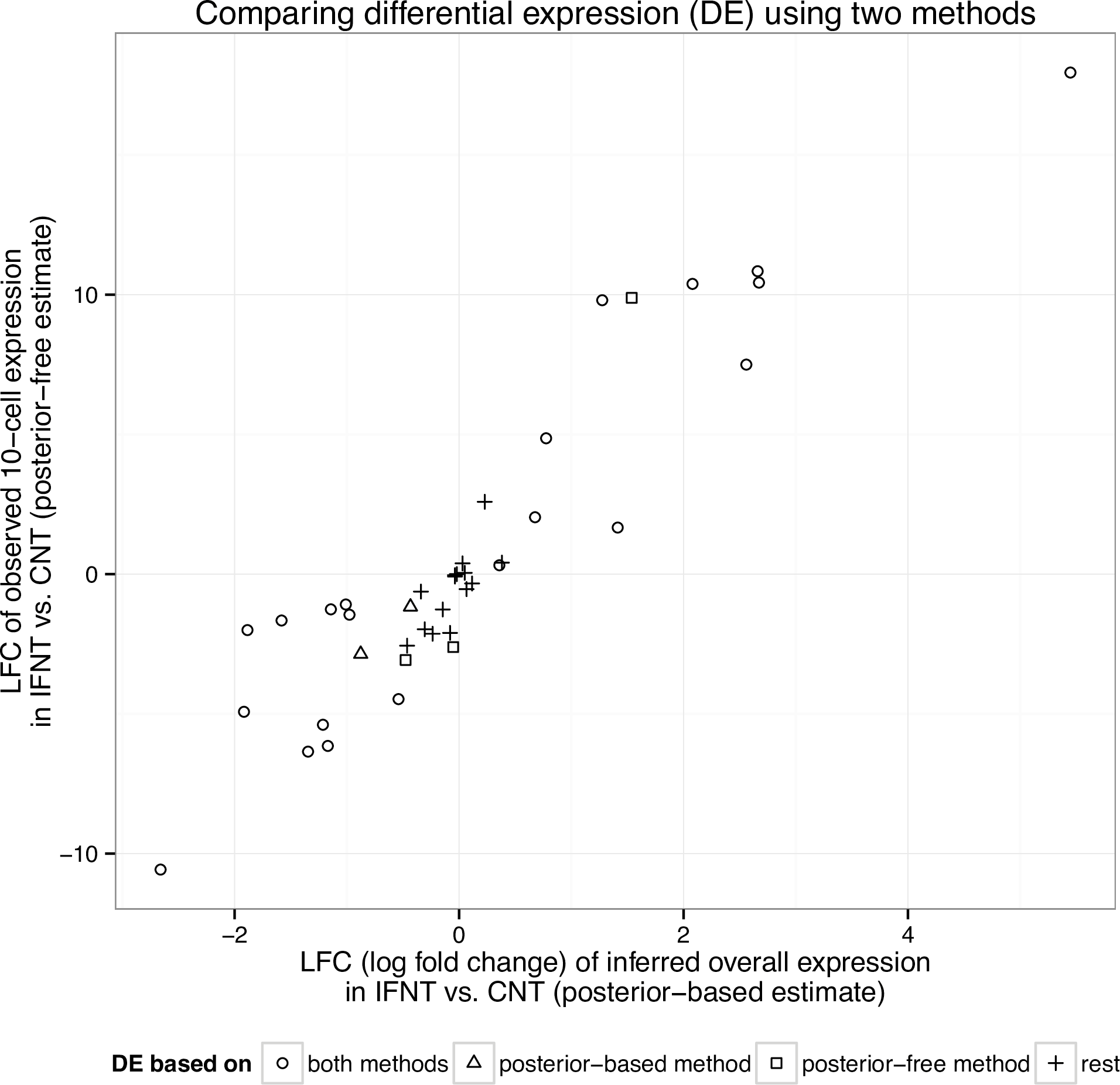

Supplement: S8 Fig — For the genes successfully modeled in both IFNT and CNT conditions by our approach, we show the log2 fold change of their bulk/overall mean expression between the two conditions and the list of significant differentially expressed (DE) genes at adjusted P < 0.05, as determined by two methods. Though the DE results from both methods are not expected to be exactly equal (as explained in Methods), the concordance between them as shown here reinforces each method’s discoveries. (TIF) [file pcbi.1005016.s008.tif]

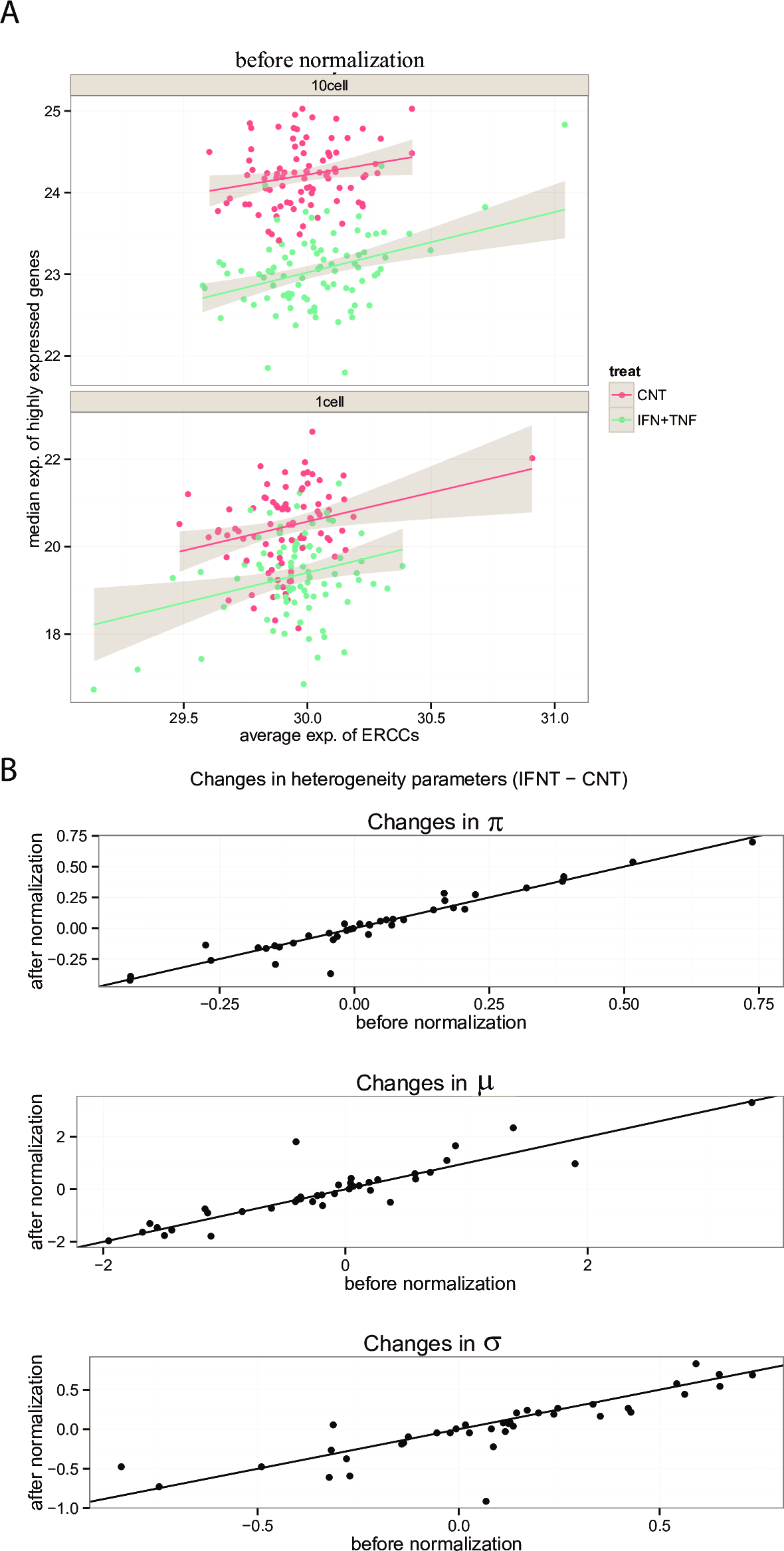

Supplement: S9 Fig — We augmented our method with an additional pre-processing/normalization step that accounts for one of the key noise sources: well-to-well variation in sampling efficiency (inspired by the “Model 1” strategy used in (Grün et al. 2014, Nat. Methods 11, 637–640 [12])). This normalization involved inferring this noise using the Et values of three ERCC control mRNAs spiked in at the same concentration across wells, and then removing the effect of this noise from the data and applying our Bayesian inference procedure on the corrected data. A. Technical factors contributed to well-to-well variations. The mean Et value of the three control ERCC spike-in mRNAs is significantly correlated with the median of highly expressed genes across wells, both within and across plates, suggesting that well-to-well differences in the starting amount of mRNA, or sampling efficiency, or other related technical factors likely contributed substantially to well-to-well variations. This plot also shows that the magnitude of this particular noise is not large in our data. Here a highly expressed gene is defined as a gene with non-zero expression level in at least 90% of all single/k-cell samples measured in either condition. Also shown are robust linear regression fits along with 95% confidence level bands (visualized using R package ggplot2’s stat_smooth function with “rlm” method and default options). B. The changes in parameter estimates between IFNT vs. CNT are largely similar before or after performing the new per-well normalization designed to remove well-to-well variations shown in (A) (the black line is the x = y diagonal line). This comparison is done using the 39 overlapping genes out of the 41 and 47 genes passing model assessment (in both CNT and IFNT conditions) in our macrophage data before and after performing the per-well normalization respectively. Note that the per-well normalization factor subtracted from each gene measurement within a well is a weighted sum of the three ERCC [file pcbi.1005016.s009.tif]

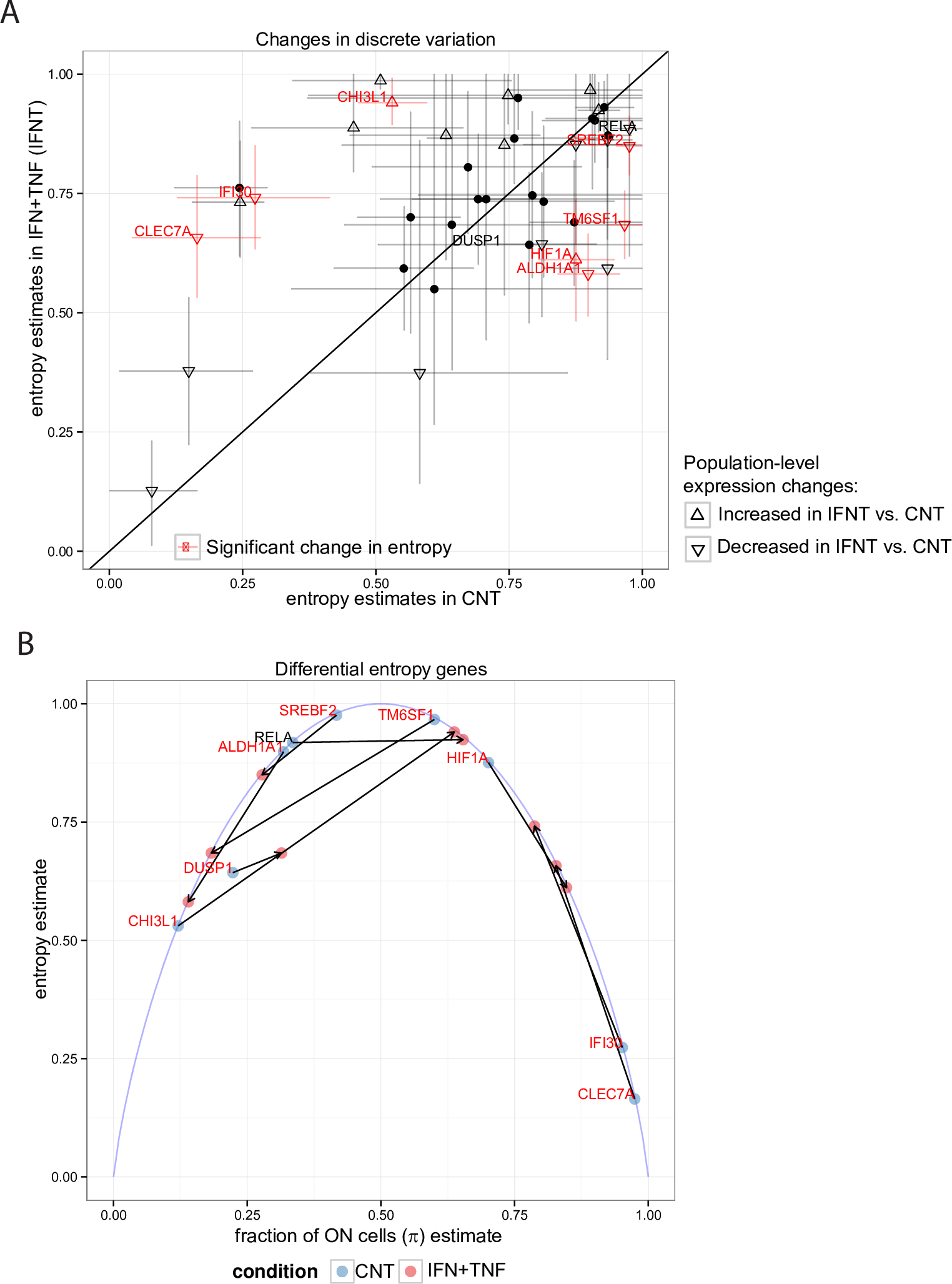

Supplement: S10 Fig — The Shannon entropy function computed using π (entropy(π) = −π log2(π) − (1 − π)log2(1 − π)) captures the intuitive notion that a cell population containing a balanced number of ON and OFF cells is more heterogeneous than another where almost all cells are ON or almost all are OFF. A. Inferred alterations in entropy in IFNT treatment relative to CNT is shown for the genes successfully modeled in both conditions. The 23 differentially expressed genes identified using overall expression changes, along with whether they are increased or decreased, is also indicated. The 90% Crl is indicated by the lightly shaded lines around the mean of the posterior distribution. B. Genes with significant (adjP < 0.05) changes in entropy between the conditions (and gene RELA) are shown along the entropy function’s curve. (TIF) [file pcbi.1005016.s010.tif]

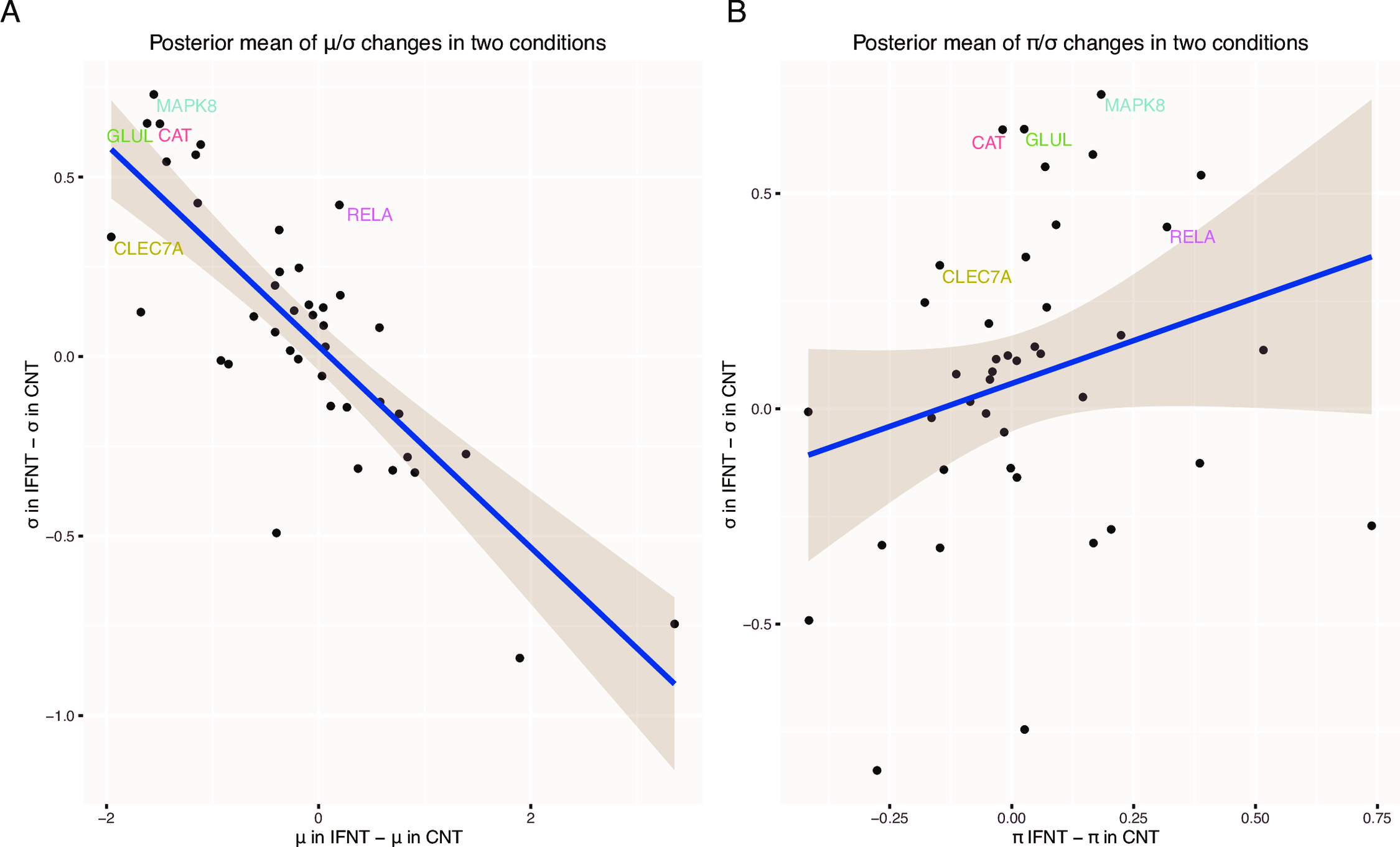

Supplement: S11 Fig — Linear regression fit of changes in continuous variation (σ) as a function of changes in the average expression among ON cells (µ; A) or ON rate (π; B) between IFNT and CNT conditions using the genes successfully modeled in both conditions. For genes with significant alterations in σ (from main text Fig 6A and labeled here), changes in σ cannot simply be explained by differences in μ or π between the conditions. Confidence bands around the linear regression fits are at 95% confidence level (and visualized using R package ggplot2’s stat_smooth function with “lm” method and default options). (TIF) [file pcbi.1005016.s011.tif]

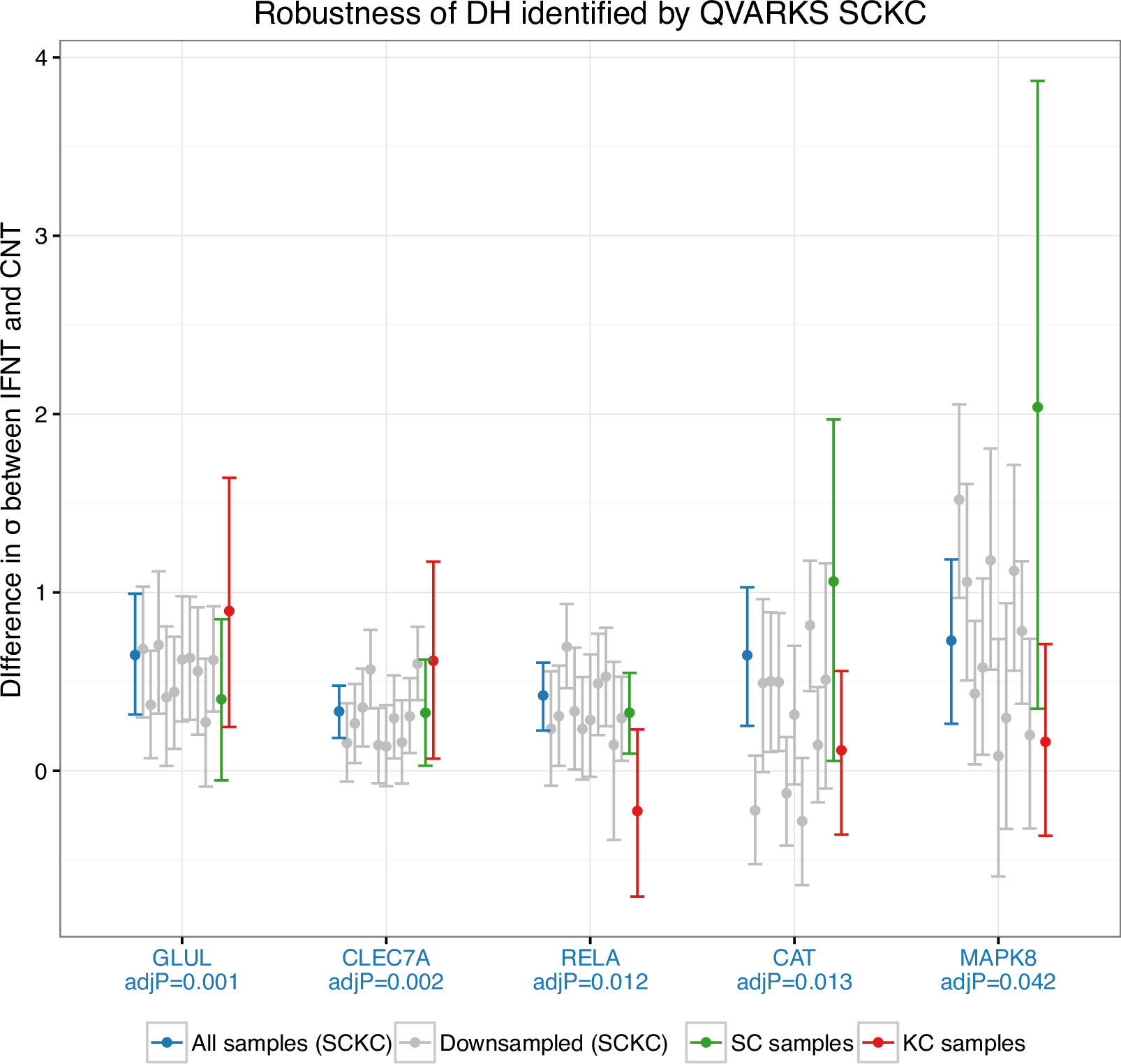

Supplement: S12 Fig — For genes with significant alterations in σ (DH genes from main text Fig 6A, with adjusted Pvalues reported here), difference in σ was non-zero for many of the ten random 50% downsamplings of the dataset (the same downsamplings as in main text Fig 3). Also shown here for comparison are the inferences made by QVARKS on two non-overlapping subsets of the data: single-cell (SC) samples alone vs. k-cell (KC) samples alone. The 90% Crl is indicated by the line around the mean of the posterior distribution. (TIF) [file pcbi.1005016.s012.tif]
